# Efficacy and safety of filgotinib in patients with rheumatoid arthritis: week 156 interim results from a long-term extension study

**DOI:** 10.1136/rmdopen-2024-004476

**Published:** 2024-10-24

**Authors:** Maya H Buch, Daniel Aletaha, Bernard G Combe, Yoshiya Tanaka, Roberto Caporali, Hendrik Schulze-Koops, Tsutomu Takeuchi, Jacques-Eric Gottenberg, Ricardo Blanco, Patrick Verschueren, Anna Zubrzycka-Sienkiewicz, Francesco De Leonardis, Edmund V Ekoka Omoruyi, Vijay Rajendran, Paul Emery

**Affiliations:** 1 Centre for Musculoskeletal Research, University of Manchester, Manchester, UK; 2 NIHR Manchester Biomedical Research Centre, Manchester, UK; 3 Division of Rheumatology, Medical University of Vienna, Vienna, Austria; 4 Rheumatology, Montpellier University, Montpellier, Occitanie, France; 5 University of Occupational and Environmental Health Japan, Kitakyushu, Japan; 6 University of Milan, Milan, Italy; 7 ASST Gaetano Pini-CTO, Milan, Italy; 8 Division of Rheumatology and Clinical Immunology, Department of Internal Medicine IV, Ludwig Maximilian University of Munich, Munich, Germany; 9 Keio University, Tokyo, Japan; 10 Saitama Medical University, Saitama, Japan; 11 Rheumatology, Strasbourg University Hospitals, Strasbourg, Grand Est, France; 12 Immunopathology group, Hospital Universitario Marqués de Valdecilla, IDIVAL, Santander, Cantabria, Spain; 13 Department of Rheumatology, KU Leuven and University Hospital Leuven, Leuven, Belgium; 14 ARS Rheumatica, Warsaw, Poland; 15 Galapagos GmbH, Basel, Switzerland; 16 Galapagos NV, Mechelen, Belgium; 17 University of Leeds, Leeds, UK; 18 NIHR Leeds Biomedical Research Centre, Leeds, UK

**Keywords:** antirheumatic agents, arthritis, rheumatoid, Therapeutics

## Abstract

**Background:**

Janus kinase inhibitors are an effective option for achieving sustained remission or low disease activity in patients with rheumatoid arthritis (RA) following inadequate response to conventional synthetic disease-modifying anti-rheumatic drugs. Filgotinib is a Janus kinase 1–preferential inhibitor available in two doses for moderate-to-severe RA. We report the long-term efficacy and safety of filgotinib.

**Methods:**

In the ongoing long-term extension study FINCH 4 (NCT03025308), patients continue filgotinib 200 mg or 100 mg from FINCH 1, 2 or 3 or receive filgotinib 200 mg or 100 mg de novo. Efficacy assessments up to week 156 include American College of Rheumatology 20% response (ACR20), Disease Activity Score 28 using C-reactive protein of <2.6, Clinical Disease Activity Index of ≤2.8, Simplified Disease Activity Index of ≤3.3 and Boolean remission (1.0 and 2.0) with non-responder imputation.

**Results:**

In patients with an inadequate response to methotrexate, 60.2% and 54.6% receiving de novo filgotinib 200 mg and 100 mg had an ACR20 at week 156, respectively, as did 67.3% and 59.5% of those who continued filgotinib 200 mg and 100 mg. At week 156, Boolean remission 1.0 was achieved by 18.8% and 15.4% of patients treated with de novo filgotinib 200 mg and 100 mg, respectively, and by 21.1% and 18.5% when Boolean 2.0 criteria were applied. Similar efficacy data were seen in patients from FINCH 2 and 3. Safety data were consistent with the known safety profile of filgotinib.

**Conclusion:**

In FINCH 4, filgotinib 200 mg and 100 mg (continuous or de novo) demonstrated sustained efficacy up to week 156 in patients enrolled from FINCH 1, 2 or 3, with no unexpected safety results.

**WHAT IS ALREADY KNOWN ON THIS TOPIC**

- The preferential Janus kinase 1 inhibitor filgotinib demonstrated efficacy and was generally well tolerated in patients with rheumatoid arthritis (RA) in phase 3 randomised controlled trials (FINCH 1–3).

**WHAT THIS STUDY ADDS**

- Results from the long-term extension study FINCH 4 showed filgotinib 200 mg and 100 mg continued to demonstrate efficacy, as assessed by a range of measures including American College of Rheumatology 20%/50%/70% responses, Clinical Disease Activity Index, Disease Activity Score 28 using C-reactive protein, Simplified Disease Activity Index and Boolean 1.0 and 2.0 remission. The safety data observed during the long-term extension were in line with the known safety profile for filgotinib.

**HOW THIS STUDY MIGHT AFFECT RESEARCH, PRACTICE OR POLICY**

- These findings provide clinicians with evidence that filgotinib can continue to provide long-term clinical benefits to patients with RA.

## Introduction

 Rheumatoid arthritis (RA) is a chronic autoimmune disease characterised by inflammation of the joints, substantial pain and decreased quality of life.^1 2^ Current recommendations advocate a treat-to-target approach, whereby treatment is adjusted until the treatment goal—usually sustained remission or low disease activity—is achieved.^3 4^ The initial treatment with conventional synthetic (cs) disease-modifying anti-rheumatic drugs (DMARDs), such as methotrexate, is recommended as soon as the diagnosis is confirmed. If the treatment target is not reached, depending on the patient’s risk profile, other csDMARDs should be considered or a biologic (b) DMARD or targeted synthetic (ts) DMARD included.^3 4^

Filgotinib is a preferential Janus kinase (JAK) 1 inhibitor, available in two doses (100 mg and 200 mg daily), for the treatment of moderate-to-severe RA in adult patients who have responded inadequately or are intolerant to one or more csDMARDs.^5^ Filgotinib was evaluated in FINCH 1–3 studies, which were phase 3 randomised controlled trials conducted in methotrexate-naïve patients (FINCH 3) and in those with an inadequate response to methotrexate (FINCH 1) or bDMARDs (FINCH 2).^6^,^8^ Each study met its primary endpoint by demonstrating that a significantly greater proportion of patients treated with filgotinib achieved American College of Rheumatology 20% response (ACR20) compared with those treated with either placebo at week 12 (FINCH 1 and 2) or methotrexate at week 24 (FINCH 3).^6^,^8^ In addition, other endpoints associated with signs and symptoms of RA improved with filgotinib treatment.^6^,^8^ In the RA clinical trial programme, filgotinib was generally well tolerated.^9^ To evaluate the long-term efficacy and safety of filgotinib, patients completing treatment in FINCH 1, 2 or 3 could participate in FINCH 4, a long-term extension study. In the current analysis, we report interim efficacy, safety and patient-reported outcomes up to week 156 of FINCH 4. Given that the Boolean 2.0 criteria were recently validated,^10^ Boolean 2.0 remission is reported as an exploratory objective. Further data will be reported upon study completion.

## Methods

### Study design

FINCH 4 (NCT03025308) is an ongoing, phase 3, open-label, long-term extension study. The primary outcome is safety and tolerability; the secondary outcome is efficacy. Eligible patients are adults with RA who completed one of the previous phase 3 randomised controlled trials of filgotinib: FINCH 1 (NCT02889796), FINCH 2 (NCT02873936) or FINCH 3 (NCT02886728). As previously reported, FINCH 1 was a 52-week study in which patients with an inadequate response to methotrexate (methotrexate-IR) received filgotinib 100 mg, filgotinib 200 mg, adalimumab or placebo, each with methotrexate.^6^ FINCH 2 was a 24-week study in which patients with an inadequate response to bDMARDs (bDMARD-IR) received filgotinib 100 mg, filgotinib 200 mg or placebo (each with one or two protocol-specified csDMARDs).^7^ FINCH 3 was a 52-week study in which patients who were methotrexate naïve received filgotinib 200 mg, methotrexate or filgotinib 100 mg or 200 mg with methotrexate.^8^ In FINCH 4, patients are being treated with filgotinib 100 mg or 200 mg for up to 6 years (online supplemental figure 1). In FINCH 4, patients can continue to receive csDMARDs that were permitted in the parent study, with the exception of patients from FINCH 3, who undergo a 4-week methotrexate wash-out period before inclusion in FINCH 4.

The trial was conducted in accordance with the Declaration of Helsinki and International Council for Harmonisation Good Clinical Practice guidelines. FINCH 1 was approved by the Advarra Central Institutional Review Board (Reference # 00000971). FINCH 2 was approved by the Administrative Panel on Human Subjects in Medical Research (Reference # 4593). FINCH 3 was approved by the Ethics Committee Research UZ/KU Leuven (Reference # S59627). The study protocol was approved by the international review board or ethics committee at each study site, and all patients provided written informed consent.

### Assessments

Efficacy of filgotinib 100 mg and 200 mg was assessed according to previous filgotinib exposure in the parent studies: patients either received de novo filgotinib in FINCH 4, having been re-randomised to filgotinib from adalimumab in FINCH 1, placebo in FINCH 2 or methotrexate in FINCH 3 or continued to receive filgotinib in FINCH 4, having been treated in a filgotinib arm during the parent study. Patients in FINCH 1, who initially received placebo and were re-randomised to filgotinib 100 mg or 200 mg at week 24, were included in the subgroup of patients who continued to receive filgotinib.

Efficacy of filgotinib 100 mg and 200 mg was assessed by measuring the proportion of patients to achieve the following outcomes at weeks 2, 6, 12 and then every 12 weeks up to week 156 of the long-term extension: ACR20, ACR50 and ACR70 (calculated using the baseline values of the parent study), Disease Activity Score 28 using C-reactive protein (DAS28-CRP) of <2.6, Clinical Disease Activity Index (CDAI) of ≤2.8, Simplified Disease Activity Index (SDAI) of ≤3.3, Boolean remission 1.0 and Boolean remission 2.0 (the threshold for patient global assessment in Boolean 2.0 remission is increased from 1 cm to 2 cm on a 10-cm visual analogue scale [VAS]^10^). In addition, changes from baseline in patient-reported pain (measured using a VAS) and Health Assessment Questionnaire–Disability Index (HAQ-DI) were assessed.

To assess the safety of filgotinib, the exposure-adjusted incidence rate (EAIR) of treatment-emergent adverse events (TEAEs) per 100 patient-years of exposure was calculated. The 95% CI of the EAIR was calculated based on Poisson distribution.^11^ EAIRs of TEAEs are reported based on filgotinib dose and according to previous filgotinib exposure in parent studies. TEAEs were defined as any adverse events that began on or after the study drug start date, up to 30 days post-permanent discontinuation of the study drug. If a TEAE was reported multiple times for a patient (with different start and end dates) in the same treatment period, the onset of the first TEAE occurrence was used for EAIR analysis. The severity of TEAEs was graded using the modified Common Terminology Criteria for Adverse Events (CTCAE) version 4.03. If a CTCAE criterion did not exist, the investigator used the following grades to describe the maximum intensity of the adverse event: Grade 1 (mild), Grade 2 (moderate), Grade 3 (severe), Grade 4 (life-threatening) or Grade 5 (fatal). A serious TEAE was defined as an event resulting in death, in-patient hospitalisation or prolongation of existing hospitalisation; persistent or significant disability/incapacity; life-threatening events; a congenital anomaly/birth defect; or a medically important event or reaction that may jeopardise the patient or require intervention to prevent one of the other serious TEAEs described. The investigator or qualified sub-investigator was responsible for determining whether TEAEs were related to the study drug based on their clinical judgement.

### Statistical analysis

The primary analysis set for safety and efficacy is the safety analysis set, which included all enrolled patients who received at least one dose of filgotinib in FINCH 4. Non-responder imputation (NRI) was performed for binary efficacy outcomes (ACR20, ACR50, ACR70, DAS28-CRP <2.6, CDAI ≤2.8, SDAI ≤3.3 and Boolean remission [1.0 and 2.0]), under which patients with missing outcomes were classified as non-responders. In addition, observed case (OC) analyses were performed.

## Results

### Baseline characteristics and filgotinib exposure

In total, 2729 patients were included in the analysis. As of 6 May 2022, 1723 of these patients (63.1%) were still receiving the study drug and 1006 (36.9%) had prematurely discontinued the study drug. The reasons for discontinuations are shown in online supplemental table 1. At FINCH 4 baseline, the mean (SD) age was 54 (12.9) years, mean (SD) body mass index was 27.9 (6.32) mg/kg^2^ and 80.5% of patients were female. The median (IQR) duration of RA was 4.3 (1.7–9.9) years (table 1). Approximately half of patients in FINCH 4 (51.3%) were methotrexate-IR (from FINCH 1), 13.6% were bDMARD-IR (from FINCH 2) and 35.2% were methotrexate naïve (from FINCH 3) at inclusion in the parent study. Median (IQR) exposure to filgotinib during FINCH 4 was 205.6 (162.0–229.7) weeks (online supplemental table 2).

**Table 1:** Baseline characteristics at LTE baseline

	FIL200			FIL100			Total (n=2729)
	With continued FIL (n=1195)	With de novo FIL (n=335)	Total (n=1530)	With continued FIL (n=863)	With de novo FIL (n=336)	Total (n=1199)	
Age (years), mean (SD)	53 (13.2)	54 (12.1)	53 (12.9)	54 (12.4)	55 (13.9)	54 (12.8)	54 (12.9)
Duration of RA from diagnosis (years)*							
Mean (SD)	6.7 (7.43)	6.9 (8.59)	6.7 (7.70)	8.5 (8.02)	6.8 (7.61)	8.0 (7.94)	7.3 (7.83)
Median (IQR)	3.8 (1.6–8.7)	3.5 (1.5–9.0)	3.8 (1.5–8.8)	5.7 (2.2–12.0)	4.0 (1.4–9.8)	5.1 (1.9–11.4)	4.3 (1.7–9.9)
Female, n (%)	960 (80.3)	267 (79.7)	1227 (80.2)	699 (81.0)	272 (81.0)	971 (81.0)	2198 (80.5)
Baseline BMI (kg/m^2^), mean (SD)	27.9 (6.29)	27.9 (6.42)	27.9 (6.32)	27.9 (6.40)	27.9 (6.14)	27.9 (6.32)	27.9 (6.32)
Current smoker, n (%)	160 (13.4)	48 (14.3)	208 (13.6)	119 (13.8)	39 (11.6)	158 (13.2)	366 (13.4)
Parent study, n (%)							
FINCH 1	571 (47.8)	128 (38.2)	699 (45.7)	570 (66.0)	130 (38.7)	700 (58.4)	1399 (51.3)
FINCH 2	132 (11.0)	59 (17.6)	191 (12.5)	124 (14.4)	55 (16.4)	179 (14.9)	370 (16.6)
FINCH 3	492 (41.2)	148 (44.2)	640 (41.8)	169 (19.6)	151 (44.9)	320 (26.7)	960 (35.2)
hsCRP (mg/L),mean (SD)	n=11944.90 (8.77)	n=3357.53 (11.56)	n=15295.48 (9.51)	n=8596.55 (11.20)	n=3337.50 (12.45)	n=11926.82 (11.57)	n=27216.07 (10.48)
DAS28-CRP, mean (SD)	n=11912.5 (1.08)	n=3343.0 (1.27)	n=15252.6 (1.14)	n=8552.8 (1.15)	n=3332.9 (1.24)	n=11882.8 (1.18)	n=27132.7 (1.16)
DAS28-CRP ≤3.2, n (%)	919 (77.2)	208 (62.3)	1127 (73.9)	592 (69.2)	210 (63.1)	802 (67.5)	1929 (71.1)
HAQ-DI, mean (SD)	n=11920.66 (0.62)	n=3340.81 (0.65)	n=15260.69 (0.63)	n=8610.78 (0.67)	n=3360.84 (0.69)	n=11970.80 (0.68)	n=27230.74 (0.65)
SDAI, mean (SD)	n=11907.6 (8.10)	n=33411.0 (10.82)	n=15248.4 (8.87)	n=8539.2 (8.96)	n=33310.3 (10.19)	n=11869.5 (9.33)	n=27108.9 (9.09)
CDAI, mean (SD)	n=11917.2 (7.90)	n=33410.2 (10.38)	n=15257.8 (8.59)	n=8578.5 (8.61)	n=3369.6 (9.66)	n=11938.8 (8.93)	n=27188.3 (8.75)
Patient’s pain assessment (mm),mean (SD)	n=119123 (23.0)	n=33429 (26.1)	n=152624 (23.8)	n=86126 (23.8)	n=33630 (26.8)	n=119727 (24.7)	n=272325 (24.3)
Prior exposure to bDMARDs, n (%)	146 (12.2)	186 (55.5)	332 (21.7)	134 (15.5)	185 (55.1)	319 (26.6)	651 (23.9)
To adalimumab in FINCH 1	–	128 (38.2)	128 (8.4)	–	130 (38.7)	130 (10.8)	258 (9.5)
Concurrent oral CS on first dosing date,n (%)	471 (39.4)	146 (43.6)	617 (40.3)	395 (48.8)	130 (38.7)	525 (43.8)	1142 (41.8)
Concurrent methotrexate on first dosing date, n (%)	624 (52.2)	158 (47.2)	782 (51.1)	598 (69.3)	156 (46.4)	754 (62.9)	1536 (56.3)
Number of concurrent csDMARDs on first dosing date, n (%)							
0	504 (42.2)	154 (46.0)	658 (43.0)	230 (26.7)	154 (45.8)	384 (32.0)	1042 (38.2)
1	627 (52.5)	164 (49.0)	791 (51.7)	563 (65.2)	166 (49.4)	729 (60.8)	1520 (55.7)
≥2	64 (5.4)	17 (5.1)	81 (5.3)	70 (8.1)	16 (4.8)	86 (7.2)	167 (6.1)
Race, n (%)							
American Indian or Alaska Native	82 (6.9)	25 (7.5)	107 (7.0)	58 (6.7)	27 (8.0)	85 (7.1)	192 (7.0)
Asian	251 (21.0)	60 (17.9)	311 (20.3)	186 (21.6)	60 (17.9)	246 (20.5)	557 (20.4)
Black or African American	32 (2.7)	13 (3.9)	45 (2.9)	21 (2.4)	20 (6.0)	41 (3.4)	86 (3.2)
Native Hawaiian or Pacific Islander	1 (<0.1)	0	1 (<0.1)	0	0	0	1 (<0.1)
White	817 (68.4)	233 (69.6)	1050 (68.6)	585 (67.8)	226 (67.3)	811 (67.6)	1861 (68.2)
Other	11 (0.9)	3 (0.9)	14 (0.9)	12 (1.4)	3 (0.9)	15 (1.3)	29 (1.1)
Not reported	1 (<0.1)	1 (0.3)	2 (0.1)	1 (0.1)	0	1 (<0.1)	3 (0.1)
Ethnicity, n (%)							
Hispanic or Latino	221 (18.5)	69 (20.6)	290 (19.0)	150 (17.4)	72 (21.4)	222 (18.5)	512 (18.8)
Not Hispanic or Latino	968 (81.0)	265 (79.1)	1233 (80.6)	704 (81.6)	264 (78.6)	968 (80.7)	2201 (80.7)
Not reported	6 (0.5)	1 (0.3)	7 (0.5)	9 (1.0)	0	9 (0.8)	16 (0.6)

Concurrent medication use was defined as medications taken while a patient was receiving the study drug; patients started concurrent medication prior to the first dosing. Concurrent oral corticosteroids use on as -needed frequency was excluded from the mean oral corticosteroids daily dose.

* Duration of RA (years) = (first dose date in LTE – date of initial diagnosis+1)/365.25.

bDMARDbiologic disease-modifying anti-rheumatic drugBMIbody mass indexCDAIClinical Disease Activity IndexCScorticosteroidcsDMARDconventional synthetic disease-modifying anti-rheumatic drugDAS28-CRPDisease Activity Score 28 using C-reactive proteinFIL(100/200)filgotinib (100 mg/200 mg)HAQ-DIHealth Assessment Questionnaire–Disability IndexhsCRPhigh-sensitivity C-reactive proteinLTElong-term extensionRArheumatoid arthritisSDAISimplified Disease Activity Index

### ACR responses and disease activity measures

**Figure 1:**
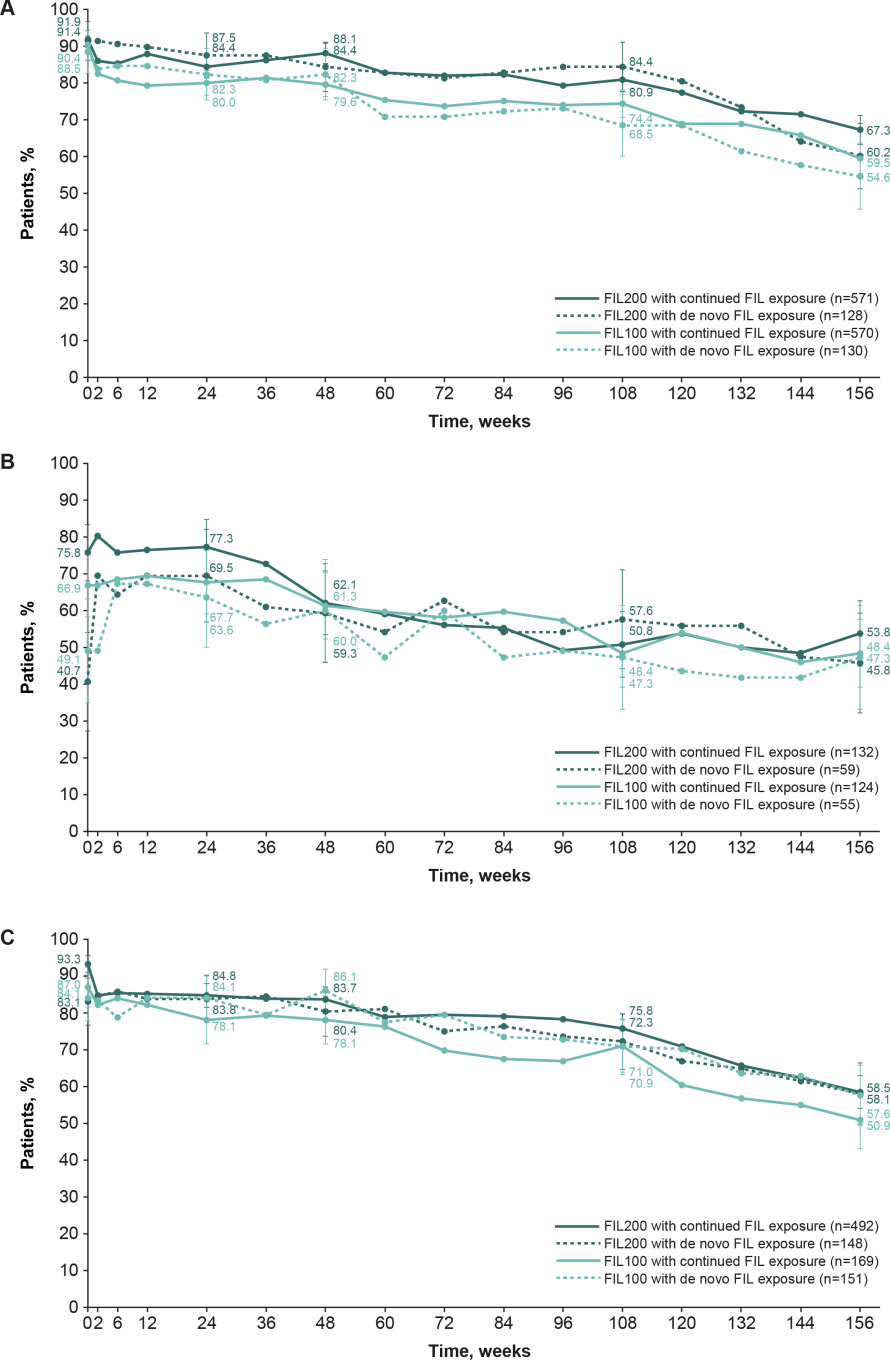
The proportion of patients who achieved ACR20 in FINCH 4 according to the parent study: FINCH 1 (**A**), FINCH 2 (**B**) and FINCH 3 (**C**) (safety analysis set, NRI). Patients with missing outcomes were set as non-responders. ACR20 was calculated based on the parent study baseline. Error bars show 95% CIs. ACR20, American College of Rheumatology 20% response; FIL(100/200), filgotinib (100 mg/200 mg); NRI, non-responder imputation.

Based on the NRI analysis, of methotrexate-IR patients (from FINCH 1) who continued to receive filgotinib 200 mg and 100 mg in FINCH 4, 91.9% and 90.4%, respectively, had an ACR20 from baseline of FINCH 1 to baseline of FINCH 4, as did 67.3% and 59.5%, respectively, from baseline of FINCH 1 to week 156 of FINCH 4. In patients who received de novo filgotinib 200 mg and 100 mg in FINCH 4, 91.4% and 88.5%, respectively, had an ACR20 from FINCH 1 baseline to FINCH 4 baseline, as did 60.2% and 54.6%, respectively, from FINCH 1 baseline to week 156 of FINCH 4 (figure 1A). Based on the OC analysis, 93.0% and 84.3% of patients who continued to receive filgotinib 200 mg and 100 mg had an ACR20 at week 156, as did 90.6% and 85.5% of the filgotinib 200 mg and 100 mg de novo group, respectively (online supplemental figure 2A).

The NRI analysis showed that of bDMARD-IR patients (from FINCH 2) who continued to receive filgotinib 200 mg and 100 mg in FINCH 4, 75.8% and 66.9%, respectively, had an ACR20 from baseline of FINCH 2 to baseline of FINCH 4, decreasing to 53.8% and 48.4%, respectively, from baseline of FINCH 2 to week 156 of FINCH 4. In patients who received placebo in FINCH 2 and received de novo filgotinib 200 mg and 100 mg in FINCH 4, 40.7% and 49.1%, respectively, had an ACR20 from FINCH 2 baseline to FINCH 4 baseline, as did 45.8% and 47.3%, respectively, from FINCH 2 baseline to week 156 of FINCH 4 (figure 1B). Based on the OC analysis, 89.9% and 83.3% of patients who continued to receive filgotinib 200 mg and 100 mg, respectively, had an ACR20 at week 156, as did 73.0% and 70.3% of the filgotinib 200 mg and 100 mg de novo group, respectively (online supplemental figure 2B).

According to NRI analysis, in methotrexate-naïve patients (from FINCH 3) the proportion of patients with ACR20 from FINCH 3 baseline decreased from FINCH 4 baseline to week 156 in those continuing filgotinib treatment (93.3% to 58.5% for filgotinib 200 mg; 87.0% to 50.9% for filgotinib 100 mg) and in the de novo group (83.1% to 58.1% for filgotinib 200 mg; 84.1% to 57.6% for filgotinib 100 mg; figure 1C). Based on the OC analysis, 93.8% and 86.9% of patients who continued to receive filgotinib 200 mg and 100 mg, respectively, had an ACR20 at week 156, as did 87.8% and 87.9% of the filgotinib 200 mg and 100 mg de novo group, respectively (online supplemental figure 2C). Similar trends were observed when ACR50 and ACR70 were assessed in the NRI analysis (online supplemental figures 3 and 4) and OC analysis (online supplemental figures 5 and 6).

**Figure 2:**
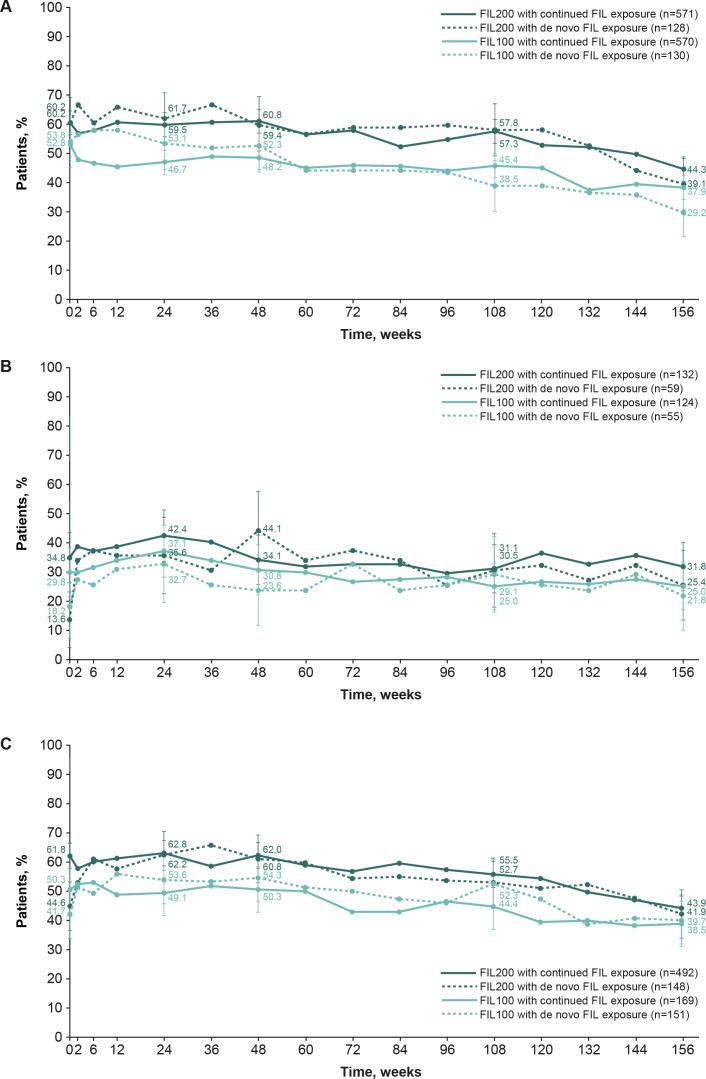
The proportion of patients who achieved DAS28-CRP of <2.6 in FINCH 4 according to the parent study: FINCH 1 (**A**), FINCH 2 (**B**) and FINCH 3 (**C**) (safety analysis set, NRI). Patients with missing outcomes were set as non-responders. DAS28-CRP was calculated based on the parent study baseline. Error bars show 95% CIs. DAS28-CRP, Disease Activity Score 28 using C-reactive protein; FIL(100/200), filgotinib (100 mg/200 mg); NRI, non-responder imputation.

Of methotrexate-IR patients who continued to receive filgotinib 200 mg and 100 mg in FINCH 4, 60.2% and 52.8%, respectively, had achieved DAS28-CRP of <2.6 at FINCH 4 baseline (NRI analysis), as had 44.3% and 37.9% respectively, at week 156 of FINCH 4. In those who received de novo filgotinib 200 mg and 100 mg, 60.2% and 53.8%, respectively, achieved DAS28-CRP of <2.6 at FINCH 4 baseline, as did 39.1% and 29.2%, respectively, at week 156 (figure 2A). Of bDMARD-IR patients who continued to receive filgotinib 200 mg and 100 mg in FINCH 4, 34.8% and 29.8%, respectively, achieved DAS28-CRP of <2.6 at FINCH 4 baseline, as did 31.8% and 25.0%, respectively, at week 156. Of those receiving de novo filgotinib 200 mg and 100 mg, 13.6% and 18.2% achieved DAS28-CRP of <2.6 at FINCH 4 baseline, as did 25.4% and 21.8%, respectively, at week 156 (figure 2B). In methotrexate-naïve patients who continued to receive filgotinib 200 mg and 100 mg, 61.8% and 50.3% achieved DAS28-CRP of <2.6 at baseline of FINCH 4, respectively, as did 43.9% and 38.5% at week 156. In those who received de novo filgotinib 200 mg and 100 mg, 44.6% and 41.7%, respectively, achieved DAS28-CRP of <2.6 at FINCH 4 baseline, as did 41.9% and 39.7%, respectively, at week 156 (figure 2C). The proportion of patients to achieve DAS28-CRP of <2.6, based on the OC analysis, is presented in online supplemental figure 7.

**Figure 3:**
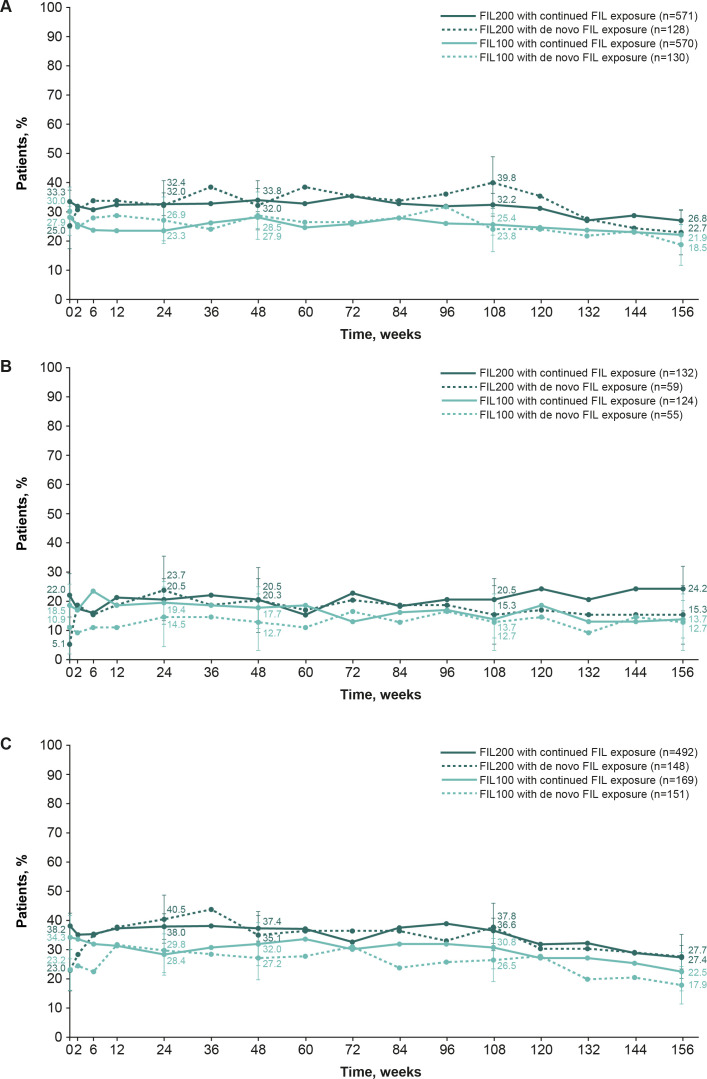
The proportion of patients who achieved CDAI of ≤2.8 in FINCH 4 according to the parent study: FINCH 1 (**A**), FINCH 2 (**B**) and FINCH 3 (**C**) (safety analysis set, NRI). Patients with missing outcomes were set as non-responders. Error bars show 95% CIs. CDAI, Clinical Disease Activity Index; FIL(100/200), filgotinib (100 mg/200 mg); NRI, non-responder imputation.

In general, the proportion of patients achieving CDAI of ≤2.8 or SDAI of ≤3.3 remained constant in each subgroup (those continuing filgotinib and those receiving de novo filgotinib) from FINCH 4 baseline to week 156 of FINCH 4, for each patient population (those from FINCH 1, 2 and 3); proportions were numerically slightly greater in the filgotinib 200 mg arm than in the filgotinib 100 mg arm in the NRI analyses (figure 3 and online supplemental figure 8) and OC analyses (online supplemental figures 9 and 10). For example, based on the NRI analysis, of methotrexate-IR patients who continued to receive filgotinib 200 mg and 100 mg in FINCH 4, 26.8% and 21.9%, respectively, achieved CDAI of ≤2.8 (remission) at week 156 (37.2% and 31.0%, respectively, in the OC analysis), as did 22.7% and 18.5%, respectively, of those receiving de novo filgotinib (33.7% and 29.3%, respectively, in the OC analysis (figure 3A and online supplemental figure 9A).

### Patient-reported outcomes: pain and HAQ-DI

In methotrexate-IR patients, change from baseline (of the parent study) in pain and HAQ-DI remained constant to week 156 of FINCH 4 and was similar for both filgotinib doses and across subgroups (those continuing filgotinib vs those receiving de novo filgotinib [online supplemental figures 11A and 12A]). Among bDMARD-IR patients, in those receiving de novo filgotinib, pain and HAQ-DI improved from FINCH 4 baseline to week 156; in those continuing to receive filgotinib, improvements in pain and HAQ-DI remained stable from FINCH 4 baseline to week 156 (online supplemental figures 11B and 12B). In methotrexate-naïve patients, changes from FINCH 4 baseline to week 156 were stable across subgroups (online supplemental figure 11C and 12C).

### Boolean remission

**Figure 4:**
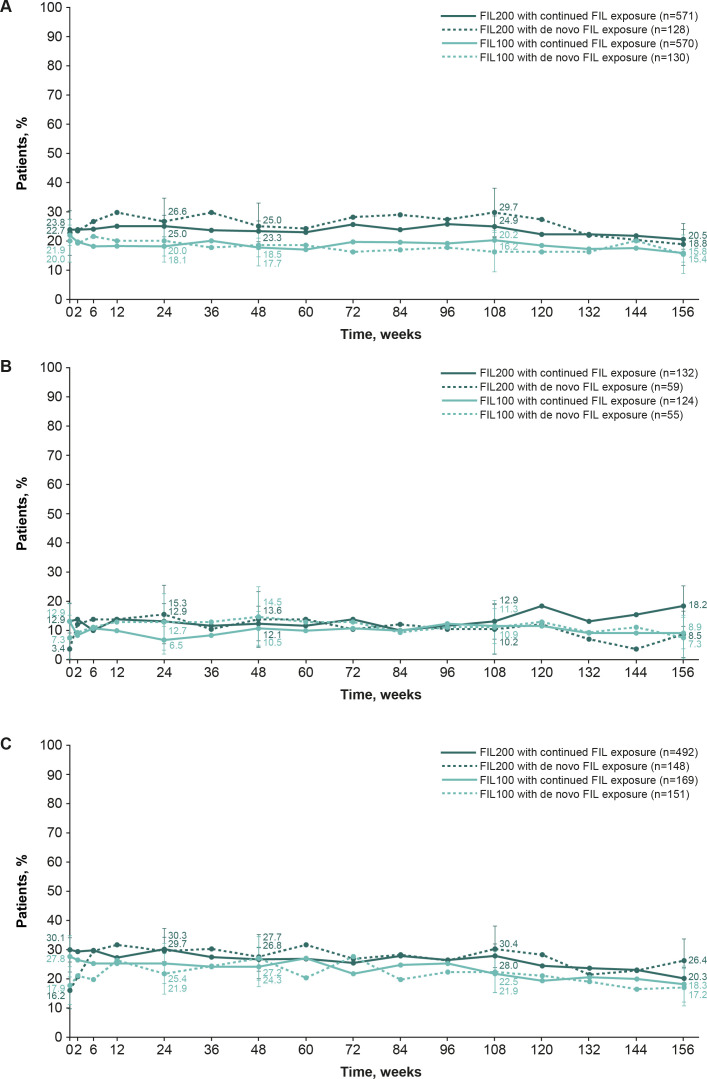
The proportion of patients who achieved Boolean remission 1.0 in FINCH 1 (**A**), FINCH 2 (**B**) and FINCH 3 (**C**) (safety analysis set, NRI). Patients with missing outcomes were set as non-responders. Error bars show 95% CIs. FIL(100/200), filgotinib (100 mg/200 mg); NRI, non-responder imputation.

In methotrexate-IR patients who continued filgotinib, the proportions achieving Boolean 1.0 remission at FINCH 4 baseline were 23.8% and 21.9% in the filgotinib 200 mg and 100 mg groups, respectively (NRI analysis). The proportions remained constant over the long-term extension, decreasing to 20.5% and 15.8% in the filgotinib 200 mg and 100 mg groups, respectively, at week 156 (NRI analysis; figure 4A). Similarly, the proportion of bDMARD-IR or methotrexate-naïve patients continuing filgotinib treatment (from FINCH 2 and 3, respectively), who achieved Boolean remission 1.0 at FINCH 4 baseline, generally remained constant up to week 156 for each filgotinib dose (NRI analysis; figure 4B,C).

**Figure 5:**
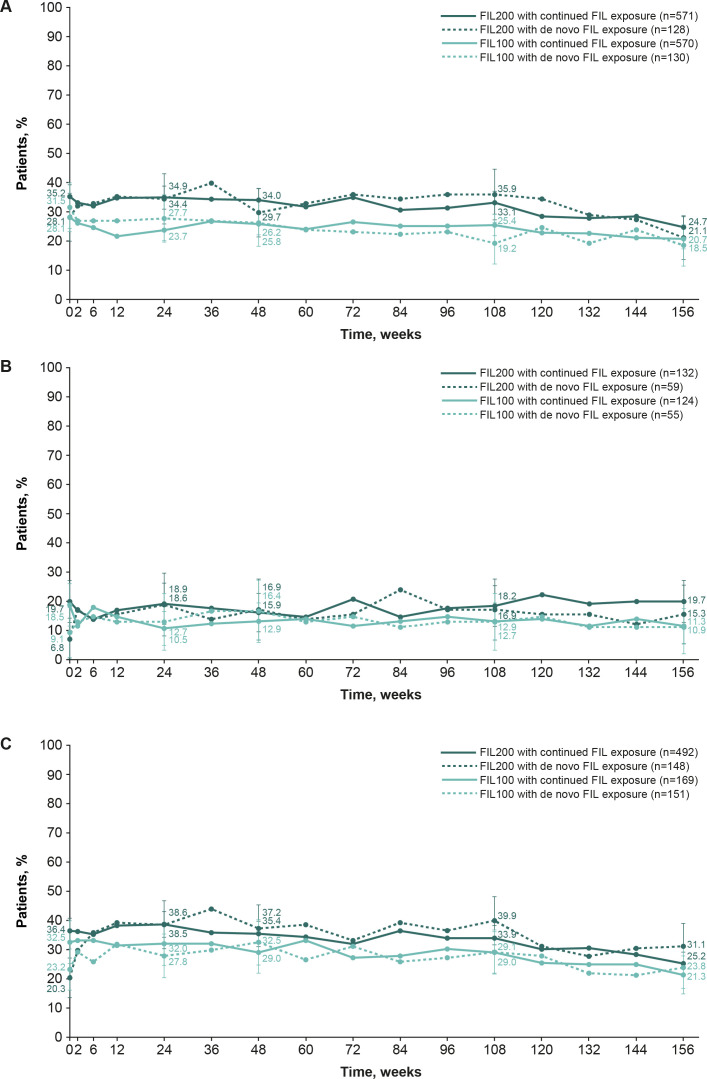
The proportion of patients who achieved Boolean remission 2.0 in FINCH 1 (**A**), FINCH 2 (**B**) and FINCH 3 (**C**) (safety analysis set, NRI). Patients with missing outcomes were set as non-responders. Error bars show 95% CIs. FIL(100/200), filgotinib (100 mg/200 mg); NRI, non-responder imputation.

Adopting Boolean 2.0 criteria slightly increased remission rates versus Boolean 1.0 criteria: for patients who continued filgotinib 200 mg and 100 mg, respectively, remission rates at week 156 increased by 4.2% and 4.9% in methotrexate-IR patients (figure 5A), by 1.5% and 2.4% in bDMARD-IR patients (figure 5B) and by 4.9% and 3.0% in methotrexate-naïve patients (figure 5C), as assessed using NRI.

In patients receiving de novo filgotinib in FINCH 4, remission rates were also numerically higher with Boolean 2.0 versus 1.0 criteria, and remission rates (using Boolean 1.0 or 2.0) were sustained up to week 156 of the long-term extension in all three patient populations (figures4  5). Sustained remission rates were also observed in the OC analyses and were numerically higher with Boolean 2.0 versus 1.0 criteria (online supplemental figures 13 and 14).

### Safety

Safety data were obtained from 1530 patients with a total of 4591.2 patient-years of exposure (PYE) to filgotinib 200 mg (from FINCH 4 baseline) and 1199 patients with a total of 3553.8 PYE to filgotinib 100 mg (from FINCH 4 baseline). In the filgotinib 200 mg and 100 mg groups, respectively, the EAIR per 100 PYE (95% CI) was 93.7 (88.7–98.9) and 91.3 (85.8–97.1) for TEAEs; 7.0 (6.3–7.9) and 7.1 (6.2–8.1) for serious TEAEs; and 0.8 (0.6–1.2) and 0.5 (0.3–0.8) for TEAEs leading to death (table 2). The TEAEs leading to death are presented in online supplemental table 3, and the TEAEs leading to premature discontinuation of the study drug are provided in online supplemental table 4. The EAIRs of TEAEs of interest, which comprised serious infections, herpes zoster, adjudicated major adverse cardiovascular events (MACE), adjudicated venous thromboembolic events, malignancies excluding non-melanoma skin cancer (NMSC) and NMSC, were comparable across treatment groups, regardless of prior exposure to filgotinib (table 2). When individual events within each of these categories were analysed, no discernible pattern was observed. However, data suggested that events within the serious infections and NMSC categories were mainly driven by COVID-19 and basal carcinoma, respectively. The most common TEAEs occurring in ≥5% of patients in either the filgotinib 200 mg or 100 mg groups are listed in table 2.

**Table 2:** Incidence and EAIR per 100 PYE of TEAEs in FINCH 4

	FIL200Number of patients (%) with the TEAE listedEAIR per 100 PYE (95% CI)			FIL100Number of patients (%) with the TEAE listedEAIR per 100 PYE (95% CI)		
	With continued FIL PYE=3572.7(n=1195)	With de novo FIL PYE=1018.5(n=335)	TotalPYE=4591.2(n=1530)	With continued FIL PYE=2568.5(n=863)	With de novo FIL PYE=985.2(n=336)	TotalPYE=3553.8(n=1199)
TEAE	1030 (86.19)93.9 (88.3 to 99.9)	293 (87.46)92.8 (82.4 to 104.0)	1323 (86.47)93.7 (88.7 to 98.9)	743 (86.10)96.0 (89.3 to 103.2)	274 (81.55)80.4 (71.2 to 90.6)	1017 (84.82)91.3 (85.8 to 97.1)
Grade ≥3 TEAE	268 (22.43)8.2 (7.3 to 9.3)	68 (20.30)7.4 (5.7 to 9.3)	336 (21.96)8.1 (7.2 to 9.0)	193 (22.36)8.3 (7.2 to 9.6)	65 (19.35)7.1 (5.5 to 9.1)	258 (21.52)8.0 (7.0 to 9.0)
Serious TEAE	237 (19.83)7.1 (6.2 to 8.1)	63 (18.81)6.7 (5.2 to 8.6)	300 (19.61)7.0 (6.3 to 7.9)	165 (19.12)6.9 (5.9 to 8.1)	68 (20.24)7.5 (5.8 to 9.5)	233 (19.43)7.1 (6.2 to 8.1)
TEAE related to study drug	394 (32.97)14.1 (12.8 to 15.6)	116 (34.63)14.5 (12.0 to 17.4)	510 (33.33)14.2 (13.0 to 15.5)	261 (30.24)12.7 (11.2 to 14.4)	101 (30.06)13.2 (10.8 to 16.0)	362 (30.19)12.9 (11.6 to 14.3)
Grade ≥3 TEAE related to study drug	78 (6.53)2.2 (1.8 to 2.8)	21 (6.27)2.1 (1.3 to 3.2)	99 (6.47)2.2 (1.8 to 2.7)	55 (6.37)2.2 (1.6 to 2.8)	12 (3.57)1.2 (0.6 to 2.2)	67 (5.59)1.9 (1.5 to 2.4)
Serious TEAE related to study drug	67 (5.61)1.9 (1.5 to 2.4)	16 (4.78)1.6 (0.9 to 2.6)	83 (5.42)1.8 (1.5 to 2.3)	37 (4.29)1.5 (1.0 to 2.0)	10 (2.98)1.0 (0.5 to 1.9)	47 (3.92)1.3 (1.0 to 1.8)
TEAE leading to interruption of study drug	484 (50.5)18.5 (16.9 to 20.2)	123 (36.72)16.1 (13.4 to 19.3)	607 (39.67)17.9 (16.5 to 19.4)	304 (35.23)15.5 (13.8 to 17.4)	132 (39.29)18.1 (15.1 to 21.5)	436 (36.36)16.2 (14.7 to 17.8)
TEAE leading to premature discontinuation of study drug	128 (10.71)3.6 (3.0 to 4.3)	36 (10.75)3.6 (2.5 to 4.9)	164 (10.72)3.6 (3.1 to 4.2)	95 (11.01)3.7 (3.0 to 4.5)	34 (10.12)3.5 (2.4 to 4.8)	129 (10.76)3.6 (3.0 to 4.3)
TEAE leading to death	34 (2.85)1.0 (0.7 to 1.3)	5 (1.49)0.5 (0.2 to 1.1)	39 (2.55)0.8 (0.6 to 1.2)	12 (1.39)0.5 (0.2 to 0.8)	7 (2.08)0.7 (0.3 to 1.5)	19 (1.58)0.5 (0.3 to 0.8)
TEAEs of interest						
Serious infection	79 (6.61)2.2 (1.8 to 2.8)	18 (5.37)1.8 (1.1 to 2.8)	97 (6.34)2.1 (1.7 to 2.6)	49 (5.68)1.9 (1.4 to 2.6)	21 (6.25)2.2 (1.3 to 3.3)	70 (5.84)2.0 (1.6 to 2.5)
Herpes zoster	52 (4.35)1.5 (1.1 to 2.0)	14 (4.18)1.4 (0.8 to 2.4)	66 (4.31)1.5 (1.1 to 1.9)	25 (2.90)1.0 (0.6 to 1.5)	15 (4.46)1.6 (0.9 to 2.6)	40 (3.34)1.1 (0.8 to 1.6)
MACE (adjudicated)	16 (1.34)0.4 (0.3 to 0.7)	1 (0.30)0.1 (0.0 to 0.5)	17 (1.11)0.4 (0.2 to 0.6)	10 (1.16)0.4 (0.2 to 0.7)	7 (2.08)0.7 (0.3 to 1.5)	17 (1.42)0.5 (0.3 to 0.8)
VTE (adjudicated)	8 (0.67)0.2 (0.1 to 0.4)	1 (0.30)0.1 (0.0 to 0.5)	9 (0.59)0.2 (0.1 to 0.4)	8 (0.93)0.3 (0.1 to 0.6)	1 (0.30)0.1 (0.0 to 0.6)	9 (0.75)0.3 (0.1 to 0.5)
Malignancy (excluding NMSC)	29 (2.43)0.8 (0.5 to 1.2)	13 (3.88)1.3 (0.7 to 2.2)	42 (2.75)0.9 (0.7 to 1.2)	20 (2.32)0.8 (0.5 to 1.2)	8 (2.38)0.8 (0.4 to 1.6)	28 (2.34)0.8 (0.5 to 1.1)
NMSC	19 (1.59)0.5 (0.3 to 0.8)	3 (0.90)0.3 (0.1 to 0.9)	22 (1.44)0.5 (0.3 to 0.7)	5 (0.58)0.2 (0.1 to 0.5)	3 (0.89)0.3 (0.1 to 0.9)	8 (0.67)0.2 (0.1 to 0.4)
Most common TEAEs (≥5% in either group)						
COVID-19	145 (12.13)4.2 (3.5 to 4.9)	41 (12.24)4.1 (3.0 to 5.6)	186 (12.16)4.2 (3.6 to 4.8)	80 (9.27)3.2 (2.5 to 4.0)	36 (10.71)3.7 (2.6 to 5.2)	116 (9.67)3.3 (2.8 to 4.0)
Nasopharyngitis	131 (10.96)4.0 (3.3 to 4.7)	32 (9.55)3.4 (2.3 to 4.8)	163 (10.65)3.9 (3.3 to 4.5)	105 (12.17)4.5 (3.7 to 5.5)	28 (8.33)3.0 (2.0 to 4.4)	133 (11.09)4.1 (3.4 to 4.9)
Upper respiratory tract infection	111 (9.29)3.3 (2.7 to 4.0)	40 (11.94)4.3 (3.1 to 5.9)	151 (9.87)3.5 (3.0 to 4.2)	82 (9.50)3.4 (2.7 to 4.2)	33 (9.82)3.6 (2.5 to 5.1)	115 (9.59)3.5 (2.9 to 4.2)
Rheumatoid arthritis (worsening, flare or exacerbation)	87 (7.28)2.6 (2.1 to 3.2)	29 (8.66)3.0 (2.0 to 4.3)	116 (7.58)2.7 (2.2 to 3.2)	103 (11.94)4.4 (3.6 to 5.3)	30 (8.93)3.3 (2.2 to 4.6)	133 (11.09)4.0 (3.4 to 4.8)
Urinary tract infection	103 (8.62)3.0 (2.5 to 3.7)	35 (10.45)3.7 (2.6 to 5.1)	138 (9.02)3.2 (2.7 to 3.8)	69 (8.00)2.8 (2.2 to 3.6)	30 (8.93)3.2 (2.2 to 4.6)	99 (8.26)2.9 (2.4 to 3.6)
Hypertension	71 (5.94)2.1 (1.6 to 2.6)	29 (8.66)3.0 (2.0 to 4.3)	100 (6.54)2.3 (1.8 to 2.8)	66 (7.65)2.7 (2.1 to 3.4)	28 (8.33)3.0 (2.0 to 4.4)	94 (7.84)2.8 (2.2 to 3.4)
Headache	66 (5.52)1.9 (1.5 to 2.4)	31 (9.25)3.2 (2.2 to 4.6)	97 (6.34)2.2 (1.8 to 2.7)	58 (6.72)2.4 (1.8 to 3.1)	20 (5.95)2.1 (1.3 to 3.2)	78 (6.51)2.3 (1.8 to 2.9)
Arthralgia	66 (5.52)1.9 (1.5 to 2.4)	24 (7.16)2.5 (1.6 to 3.7)	90 (5.88)2.0 (1.6 to 2.5)	66 (7.65)2.7 (2.1 to 3.4)	18 (5.36)1.9 (1.1 to 3.0)	84 (7.01)2.5 (2.0 to 3.1)
Latent tuberculosis	66 (5.52)1.9 (1.5 to 2.5)	13 (3.88)1.3 (0.7 to 2.2)	79 (5.16)1.8 (1.4 to 2.2)	45 (5.21)1.8 (1.3 to 2.4)	26 (7.74)2.8 (1.8 to 4.1)	71 (5.92)2.1 (1.6 to 2.6)
Bronchitis	75 (6.28)2.2 (1.7 to 2.8)	16 (4.78)1.6 (0.9 to 2.7)	91 (5.95)2.1 (1.7 to 2.5)	42 (4.87)1.7 (1.2 to 2.3)	14 (4.17)1.5 (0.8 to 2.5)	56 (4.67)1.6 (1.2 to 2.1)
Back pain	53 (4.44)1.5 (1.1 to 2.0)	17 (5.07)1.7 (1.0 to 2.8)	70 (4.58)1.6 (1.2 to 2.0)	43 (4.98)1.7 (1.2 to 2.3)	17 (5.06)1.8 (1.0 to 2.9)	60 (5.00)1.7 (1.3 to 2.2)

TEAEs are defined as any adverse events that began on or after the study drug start date, up to 30 days after permanent discontinuation of study drug. Only adverse events with a start date after LTE treatment start start are considered. EAIR, exposure-adjusted incidence rate; FIL(100/200), filgotinib ( mg/ mg); LTE, long-term extension; MACE, major adverse cardiovascular event; NMSC, non-melanoma skin cancer; PYE, ; TEAE, treatment-emergent adverse event; VTE, venous thromboembolism.

EAIRexposure-adjusted incidence rateFIL(100/200)filgotinib (100 mg/200 mg)LTElong-term extensionMACEmajor adverse cardiovascular eventsNMSCnon-melanoma skin cancerPYEpatient-years of exposureTEAEtreatment-emergent adverse eventVTEvenous thromboembolism

## Discussion

In the FINCH 4 long-term extension study, efficacy was maintained with both filgotinib 200 mg and 100 mg, over the first 156 weeks, in all three patient populations evaluated (methotrexate-IR, bDMARD-IR and methotrexate-naïve patients), both in those who received de novo filgotinib in FINCH 4 and in those who continued filgotinib treatment from the parent study. Similar patterns were seen with the NRI and OC analyses, with higher response rates seen in the OC analysis, as expected. These findings confirm that filgotinib is an effective treatment option for clinically relevant patient populations (those with RA who have not responded adequately or are intolerant to previous DMARDs). Although numerical differences between the two filgotinib doses were observed in the maintenance of remission, the results indicate that filgotinib 100 mg had largely comparable efficacy to the higher dose. This provides reassurance that treatment remains effective in situations where the lower dose is recommended, for example, in those aged 65 years or older, those at increased risk of venous thromboembolism, MACE and malignancy or those with moderate or severe renal impairment.^5^

In addition to measures of disease activity, such as ACR response criteria and DAS28-CRP, we assessed patient-reported outcomes, including pain, which is considered by patients to be a key target of the RA treatment.^12^ Pain in RA may result from inflammatory or non-inflammatory pathways, and those who achieve RA remission or low disease activity may continue to experience pain.^13 14^ In addition, early reduction in pain decreases the risk of the development of chronic pain through mechanisms other than nociception alone.^15^ Therefore, RA treatments would ideally result in rapid and long-lasting reductions in pain. Results from the current analysis show that improvements in pain from baseline of the parent study were generally maintained throughout FINCH 4. Further, in bDMARD-IR patients who were treated de novo with filgotinib, improvements in pain were seen from FINCH 4 baseline, as early as week 2, demonstrating a rapid effect, which is crucial for long-term pain control. These findings are consistent with a post hoc analysis of FINCH 1, 2 and 3, which demonstrated that filgotinib reduced pain from week 2, with improvements maintained throughout the studies.^16^

Efficacy was assessed using DAS28-CRP, CDAI, SDAI and Boolean 1.0 and 2.0 criteria. The proportion of patients in remission remained relatively stable over the long-term extension period. Boolean remission (1.0 and 2.0) was maintained through week 156 of FINCH 4 with both doses of filgotinib, regardless of whether filgotinib was taken de novo or continued from the parent study. The Boolean 2.0 criteria for remission were developed to address the overly stringent patient global assessment threshold in Boolean 1.0.^10^ A validation study by Studenic *et al* confirmed higher remission rates using Boolean 2.0, consistent with SDAI criteria, with no loss of predictive ability in terms of radiographic and functional outcomes.^10^ In line with these findings, results from the FINCH 4 study indicate that a higher proportion of patients was classed as being in remission when the Boolean 2.0 versus 1.0 criteria were applied, with ranges in line with those reported by Studenic *et al*.^10^ Similarly, data from the FINCH 4 study suggest that CDAI and SDAI remission rates were more closely aligned with Boolean 2.0 than Boolean 1.0 remission rates.

Safety data show that, in general, differences between doses in the EAIR of TEAEs were small, with overlapping 95% CIs. In patients taking de novo filgotinib, the EAIR for all TEAEs was numerically lower in the filgotinib 100 mg group than in the filgotinib 200 mg group. The EAIRs of TEAEs related to study drug and of TEAEs leading to death were numerically higher with filgotinib 200 mg than with filgotinib 100 mg. The EAIRs for the other TEAE categories reported and for TEAEs of interest (serious infection, herpes zoster, adjudicated MACE, adjudicated venous thromboembolic events, malignancy [excluding NMSC] and NMSC) were generally similar between filgotinib doses. Long-term safety data are of particular interest following the results of the ORAL Surveillance study, which showed that, in patients with RA aged 50 years or older with at least one additional cardiovascular risk factor, the incidence of major cardiovascular events and cancer was higher in those treated with tofacitinib than in those treated with a TNF inhibitor.^17^ Although no such safety signal was observed in the current analysis from FINCH 4, conclusions cannot be made, owing to the interim nature of the analysis. However, integrated data from the FINCH and DARWIN clinical studies have been reported from 3691 patients with 12 541 PYE to filgotinib. Data showed that, with a median (maximum) exposure of 3.8 (8.3) years, there were small numerical differences between filgotinib doses in the EAIRs of certain adverse events; the EAIRs of NMSC, herpes zoster and all-cause mortality were numerically slightly higher with filgotinib 200 mg than with filgotinib 100 mg, whereas the EAIRs of MACE and serious infections were numerically slightly higher with the lower dose; however, CIs overlapped between the groups.^18^ The integrated analysis included the long-term open-label extension study DARWIN 3. Kavanaugh *et al* reported up to 4-year efficacy and safety results from an interim analysis of DARWIN 3, with a focus on safety and adverse events, which were reported in detail.^19^

In terms of drug retention, it was observed that approximately 63% of patients remained on the study drug at the time of the analyses. TEAEs leading to an interruption in the study drug occurred in approximately 40% and 36% of patients in the filgotinib 200 mg and 100 mg groups, respectively, whereas TEAEs leading to premature discontinuation of the study drug occurred in approximately 11% in each treatment group.

There are several limitations associated with this analysis. There was no control group in the long-term extension study. In addition, long-term extension studies may be biased towards patients who respond to treatment; however, an NRI analysis was used to provide a conservative estimate of binary outcomes, classing those with missing data as non-responders. Patients were originally enrolled in randomised clinical trials with inclusion and exclusion criteria, which may not be representative of all patients in clinical practice. However, enrolling patients from FINCH 1 and 2 meant both methotrexate-IR and bDMARD-IR subgroups were included in the analysis, representing clinically relevant patient populations. Real-world efficacy data from a larger patient population will provide valuable insights beyond those obtained from clinical trials. Such real-world data will be provided by the ongoing phase 4, non-interventional FILOSOPHY study^20^ evaluating filgotinib for the treatment of RA in routine clinical practice. Another limitation is that as this is an interim analysis, not all data sets are available. Once the FINCH 4 study is complete, it will be important to interpret results based on EULAR recommendations for the reporting of long-term extension studies in rheumatology,^21^ for example, by including additional data and analyses from all patients over time, from baseline of the parent trial to the end of FINCH 4. While OC analyses have been included, NRI analyses, as the more stringent, are the focus of this interim analysis, to present a conservative approach to the reporting of efficacy data.

In conclusion, interim efficacy results from the FINCH 4 study confirm that beneficial effects of filgotinib 100 mg and 200 mg on disease activity measures were maintained up to week 156, independent of the initial background treatment (methotrexate-IR, bDMARD-IR and methotrexate-naïve groups). When Boolean 2.0 rather than Boolean 1.0 criteria were applied, remission rates were numerically higher and were more comparable with those reported using index-based criteria. Safety data observed during the long-term extension were in line with the known safety profile for filgotinib.

## supplementary material

10.1136/rmdopen-2024-004476 online supplemental file 1

10.1136/rmdopen-2024-004476 online supplemental file 2

## Data Availability

Data are available upon reasonable request.
