# Correction: Efficacy and safety of filgotinib in patients with rheumatoid arthritis: week 156 interim results from a long- term extension study

**DOI:** 10.1136/rmdopen-2024-004476corr1

**Published:** 2025-01-27

 To cite: Buch MH, Aletaha D, Combe BG, *et al*. Efficacy and safety of filgotinib in patients with rheumatoid arthritis: week 156 interim results from a long-term extension study. *RMD Open* 2024;10:e004476. doi: 10.1136/rmdopen-2024–0 04 476

An update has been made to table 1 in this article, where a typo had introduced an additional zero into a number of data points.

