# Supplementary material for: Efficacy and safety of filgotinib in patients with rheumatoid arthritis: week 156 interim results from a long-term extension study
Source: RMD Open. 2024 Oct 24;10(4):e004476. doi: 10.1136/rmdopen-2024-004476 (PMC11529685; doi:10.1136/rmdopen-2024-004476)
Supplement: online supplemental file 1 [file rmdopen-10-4-s001.pdf]

Supplementary material for:

Efficacy and safety of filgotinib in patients with rheumatoid arthritis: Week 156 interim results from a long-term extension study

Maya H Buch, Daniel Aletaha, Bernard G Combe, Yoshiya Tanaka, Roberto Caporali, Hendrik Schulze-Koops, Tsutomu Takeuchi, Jacques-Eric Gottenberg, Ricardo Blanco, Patrick Verschueren, Anna Zubrzycka-Sienkiewicz, Francesco De Leonardis, Edmund V Ekoka Omoruyi, Vijay Rajendran, Paul Emery

Supplemental table 1 Patient disposition

	Methotrexate-IR (FINCH 1)						
	FIL200			FIL100			
	With continued FIL	With de novo FIL*	Total	With continued FIL	With de novo FIL*	Total	Total
Safety analysis set	571	128	699	570	130	700	1399
On FIL	399 (69.9)	84 (65.6)	483 (69.1)	392 (68.8)	78 (60.0)	470 (67.1)	953 (68.1)
Discontinued FIL	172 (30.1)	44 (34.4)	216 (30.9)	178 (31.2)	52 (40.0)	230 (32.9)	446 (31.9)
Reason for discontinuation							
Sponsor's decision†	53 (9.3)	17 (13.3)	70 (10.0)	57 (10.0)	14 (10.8)	71 (10.1)	141 (10.1)
Adverse event	55 (9.6)	14 (10.9)	69 (9.9)	49 (8.6)	14 (10.8)	63 (9.0)	132 (9.4)
Withdrawal by patient	29 (5.1)	9 (7.0)	38 (5.4)	44 (7.7)	10 (7.7)	54 (7.7)	92 (6.6)
Physician's decision	8 (1.4)	2 (1.6)	10 (1.4)	7 (1.2)	6 (4.6)	13 (1.9)	23 (1.6)
Death	10 (1.8)	0	10 (1.4)	8 (1.4)	4 (3.1)	12 (1.7)	22 (1.6)
Lost to follow-up	8 (1.4)	0	8 (1.1)	3 (0.5)	3 (2.3)	6 (0.9)	14 (1.0)
Lack of efficacy	5 (0.9)	1 (0.8)	6 (0.9)	5 (0.9)	1 (0.8)	6 (0.9)	12 (0.9)
Pregnancy	1 (0.2)	1 (0.8)	2 (0.3)	3 (0.5)	0	3 (0.4)	5 (0.4)
Protocol deviation	2 (0.4)	0	2 (0.3)	1 (0.2)	0	1 (0.1)	3 (0.2)
Non-compliance	1 (0.2)	0	1 (0.1)	1 (0.2)	0	1 (0.1)	2 (0.1)
	**bDMARD-IR (FINCH 2)**						
	**FIL200**			**FIL100**			
	**With continued FIL**	**With de novo FIL***	**Total**	**With continued FIL**	**With de novo FIL***	**Total**	**Total**
Safety analysis set	132	59	191	124	55	179	370
On FIL	61 (46.2)	26 (44.1)	87 (45.5)	56 (45.2)	21 (38.2)	77 (43.0)	164 (44.3)
Discontinued FIL	71 (53.8)	33 (55.9)	104 (54.5)	68 (54.8)	34 (61.8)	102 (57.0)	206 (55.7)
Reason for discontinuation							
Sponsor's decision†	7 (5.3)	4 (6.8)	11 (5.8)	7 (5.6)	5 (9.1)	12 (6.7)	23 (6.2)
Adverse event	22 (16.7)	9 (15.3)	31 (16.2)	17 (13.7)	11 (20.0)	28 (15.6)	59 (15.9)
Withdrawal by patient	20 (15.2)	12 (20.3)	32 (16.8)	20 (16.1)	12 (21.8)	32 (17.9)	64 (17.3)

Physician's decision	8 (6.1)	4 (6.8)	12 (6.3)	7 (5.6)	2 (3.6)	9 (5.0)	21 (5.7)
Death	4 (3.0)	2 (3.4)	6 (3.1)	1 (0.8)	0	1 (0.6)	7 (1.9)
Lost to follow-up	2 (1.5)	1 (1.7)	3 (1.6)	7 (5.6)	3 (5.5)	10 (5.6)	13 (3.5)
Lack of efficacy	5 (3.8)	0	5 (2.6)	8 (6.5)	1 (1.8)	9 (5.0)	14 (3.8)
Pregnancy	1 (0.8)	0	1 (0.5)	0	0	0	1 (0.3)
Protocol deviation	1 (0.8)	0	1 (0.5)	1 (0.8)	0	1 (0.6)	2 (0.5)
Non-compliance	1 (0.8)	1 (1.7)	2 (1.0)	0	0	0	2 (0.5)
**Methotrexate-naïve (FINCH 3)**							
	**FIL100**			**FIL200**			
	**With continued FIL**	**With de novo FIL***	**Total**	**With continued FIL**	**With de novo FIL***	**Total**	**Total**
Safety analysis set	492	148	640	169	151	320	960
On FIL	311 (63.2)	99 (66.9)	410 (64.1)	97 (57.4)	99 (65.6)	196 (61.3)	606 (63.1)
Discontinued FIL	181 (36.8)	49 (33.1)	230 (35.9)	72 (42.6)	52 (34.4)	124 (38.8)	354 (36.9)
Reason for discontinuation							
Sponsor's decision†	56 (11.4)	15 (10.1)	71 (11.1)	19 (11.2)	13 (8.6)	32 (10.0)	103 (10.7)
Adverse event	38 (7.7)	13 (8.8)	51 (8.0)	26 (15.4)	8 (5.3)	34 (10.6)	85 (8.9)
Withdrawal by patient	43 (8.7)	7 (4.7)	50 (7.8)	17 (10.1)	18 (11.9)	35 (10.9)	85 (8.9)
Physician's decision	10 (2.0)	8 (5.4)	18 (2.8)	4 (2.4)	4 (2.6)	8 (2.5)	26 (2.7)
Death	13 (2.6)	1 (0.7)	14 (2.2)	1 (0.6)	2 (1.3)	3 (0.9)	17 (1.8)
Lost to follow-up	13 (2.6)	2 (1.4)	15 (2.3)	5 (3.0)	5 (3.3)	10 (3.1)	25 (2.6)
Lack of efficacy	2 (0.4)	1 (0.7)	3 (0.5)	0	0	0	3 (0.3)
Pregnancy	4 (0.8)	1 (0.7)	5 (0.8)	0	0	0	5 (0.5)
Protocol deviation	0	0	0	0	1 (0.7)	1 (0.3)	1 (0.1)
Non-compliance	2 (0.4)	1 (0.7)	3 (0.5)	0	1 (0.7)	1 (0.3)	4 (0.4)

Values are n (%). Safety analysis set includes enrolled patients who received at least one dose of study drug.

*Patients re-randomised to FIL in the FINCH 4 LTE from adalimumab (methotrexate-IR; FINCH 1), placebo (bDMARD-IR; FINCH 2) or methotrexate (methotrexate-naïve; FINCH 3) in the parent study.

†Of the 267 discontinuations that were due to the sponsor's decision, 211 were reported from Mexico. At that time, marketing application for filgotinib was not anticipated by the sponsor in Mexico, where alternative effective therapies were available.

bDMARD-IR, biologic disease-modifying anti-rheumatic drug inadequate responder; FIL(100/200), filgotinib (100 mg/200 mg); LTE, long-term extension; methotrexate-IR, methotrexate inadequate responder.

Supplemental table 2 Exposure to FIL in FINCH 4

	**FIL200**			**FIL100**			
	**With continued FIL**	**With de novo FIL**	**Total FIL200**	**With continued FIL**	**With de novo FIL**	**Total FIL100**	**Total**
N	1195	335	1530	863	336	1199	2729
Mean (SD)	200.3 (54.86)	157.4 (54.95)	190.9 (57.66)	197.1 (58.19)	151.9 (58.52)	184.4 (61.69)	188.1 (59.54)
Median	217.6	168.9	209.8	218.6	167.4	199.9	205.6
Q1; Q3	178.9; 233.3	131.9; 193.4	165.3; 229.7	171.6; 235.7	119.9; 190.7	160.0; 229.7	162.0; 229.7
Min; max	26; 293	1; 267	1; 293	25; 292	1; 270	1; 292	1; 293

FIL(100/200), filgotinib (100 mg/200 mg); max, maximum; min, minimum; Q, quartile.

Supplemental table 3 Incidence of TEAEs leading to death in FINCH 4

	FIL200<br>Number of patients (%) with the TEAE listed<br>EAIR per 100 PYE (95% CI)			FIL100<br>Number of patients (%) with the TEAE listed<br>EAIR per 100 PYE (95% CI)		
	**With continued FIL PYE=3572.7 (n=1195)**	**With de novo FIL PYE=1018.5 (n=335)**	**Total PYE=4591.2 (n=1530)**	**With continued FIL PYE=2568.5 (n=863)**	**With de novo FIL PYE=985.2 (n=336)**	**Total PYE=3553.8 (n=1199)**
COVID-19	9 (0.75)<br>0.3 (0.1–0.5)	2 (0.60)<br>0.2 (0.0–0.7)	11 (0.72)<br>0.2 (0.1–0.4)	3 (0.35)<br>0.1 (0.0, 0.3)	-	3 (0.25)<br>0.1 (0.0–0.2)
COVID-19 pneumonia	2 (0.17)<br>0.1 (0.0–0.2)	-	2 (0.13)<br>0.0 (0.0–0.2)	3 (0.35)<br>0.1 (0.0–0.3)	-	3 (0.25)<br>0.1 (0.0–0.2)
Death of unknown cause	3 (0.25)<br>0.1 (0.0–0.2)	-	3 (0.20)<br>0.1 (0.0–0.2)	-	-	-
Myocardial infarction	1 (0.08)<br>0.0 (0.0–0.2)	-	1 (0.07)<br>0.0 (0.0–0.1)	1 (0.12)<br>0.0 (0.0–0.2)	1 (0.30)<br>0.1 (0.0–0.6)	2 (0.17)<br>0.1 (0.0–0.2)
Respiratory failure	2 (0.17)<br>0.1 (0.0–0.2)	-	2 (0.13)<br>0.0 (0.0–0.2)	1 (0.12)<br>0.0 (0.0, 0.2)	-	1 (0.08)<br>0.0 (0.0, 0.2)
Acute myocardial infarction	2 (0.17)<br>0.1 (0.0–0.2)	-	2 (0.13)<br>0.0 (0.0–0.2)	-	-	-
Cardiac arrest	1 (0.08)<br>0.0 (0.0–0.2)	-	1 (0.07)<br>0.0 (0.0–0.1)	-	1 (0.30)<br>0.1 (0.0–0.6)	1 (0.08)<br>0.0 (0.0–0.2)
Cardiopulmonary failure	-	-	-	1 (0.12)<br>0.0 (0.0–0.2)	1 (0.30)<br>0.1 (0.0–0.6)	2 (0.17)<br>0.1 (0.0–0.2)
Cerebrovascular accident	1 (0.08)<br>0.0 (0.0–0.2)	-	1 (0.07)<br>0.0 (0.0–0.1)	-	1 (0.30)<br>0.1 (0.0–0.6)	1 (0.08)<br>0.0 (0.0–0.2)
Pneumonia	1 (0.08)<br>0.0 (0.0–0.2)	-	1 (0.07)<br>0.0 (0.0–0.1)	-	1 (0.30)<br>0.1 (0.0–0.6)	1 (0.08)<br>0.0 (0.0–0.2)
Pulmonary oedema	2 (0.17)<br>0.1 (0.0–0.2)	-	2 (0.13)<br>0.0 (0.0–0.2)	-	-	-
Acute kidney injury	1 (0.08)<br>0.0 (0.0–0.2)	-	1 (0.07)<br>0.0 (0.0–0.1)	-	-	-
Acute respiratory distress syndrome	1 (0.08)<br>0.0 (0.0–0.2)	-	1 (0.07)<br>0.0 (0.0–0.1)	-	-	-
Acute respiratory failure	1 (0.08)<br>0.0 (0.0–0.2)	-	1 (0.07)<br>0.0 (0.0–0.1)	-	-	-

	FIL200<br>Number of patients (%) with the TEAE listed<br>EAIR per 100 PYE (95% CI)			FIL100<br>Number of patients (%) with the TEAE listed<br>EAIR per 100 PYE (95% CI)		
	**With continued FIL<br>PYE=3572.7<br>(n=1195)**	**With de novo FIL<br>PYE=1018.5<br>(n=335)**	**Total<br>PYE=4591.2<br>(n=1530)**	**With continued FIL<br>PYE=2568.5<br>(n=863)**	**With de novo FIL<br>PYE=985.2<br>(n=336)**	**Total<br>PYE=3553.8<br>(n=1199)**
Aortic dissection	1 (0.08)<br>0.0 (0.0–0.2)	-	1 (0.07)<br>0.0 (0.0–0.1)	-	-	-
Brain oedema	1 (0.08)<br>0.0 (0.0–0.2)		1 (0.07)<br>0.0 (0.0–0.1)	-	-	-
Candida pneumonia	-	-	-	-	1 (0.30)<br>0.1 (0.0–0.6)	1 (0.08)<br>0.0 (0.0–0.2)
Cardiac failure	-	1 (0.30)<br>0.1 (0.0–0.5)	1 (0.07)<br>0.0 (0.0–0.1)	-	-	-
Cardiogenic shock	-	-	-	-	1 (0.30)<br>0.1 (0.0–0.6)	1 (0.08)<br>0.0 (0.0–0.2)
Diabetic hyperglycaemic coma	-	-	-	1 (0.12)<br>0.0 (0.0–0.2)	-	1 (0.08)<br>0.0 (0.0–0.2)
Electrolyte imbalance	1 (0.08)<br>0.0 (0.0–0.2)	-	1 (0.07)<br>0.0 (0.0–0.1)	-	-	-
Gastrointestinal haemorrhage	1 (0.08)_<br>0.0 (0.0–0.2)	-	1 (0.07)<br>0.0 (0.0–0.1)	-	-	-
Heat illness	-	-	-	1 (0.12)<br>0.0 (0.0–0.2)	-	1 (0.08)<br>0.0 (0.0–0.2)
Hydrothorax	1 (0.08)<br>0.0 (0.0–0.2)	-	1 (0.07)<br>0.0 (0.0–0.1)	-	-	-
Hyponatraemia	1 (0.08)<br>0.0 (0.0–0.2)	-	1 (0.07)<br>0.0 (0.0–0.1)	-	-	-
Ischaemic stroke	1 (0.08)<br>0.0 (0.0–0.2)	-	1 (0.07)<br>0.0 (0.0–0.1)	-	-	-
Lung adenocarcinoma	-	1 (0.30)<br>0.1 (0.0–0.5)	1 (0.07)<br>0.0 (0.0–0.1)	-	-	-
Lung cancer metastatic	1 (0.08)<br>0.0 (0.0–0.2)	-	1 (0.07)<br>0.0 (0.0–0.1)	-	-	-
Malignant peritoneal neoplasm	-	-	-	1 (0.12)<br>0.0 (0.0–0.2)	-	1 (0.08)<br>0.0 (0.0–0.2)
Metastatic gastric cancer	1 (0.08)<br>0.0 (0.0–0.2)	-	1 (0.07)<br>0.0 (0.0–0.1)	-	-	-

	FIL200<br>Number of patients (%) with the TEAE listed<br>EAIR per 100 PYE (95% CI)			FIL100<br>Number of patients (%) with the TEAE listed<br>EAIR per 100 PYE (95% CI)		
	**With continued FIL PYE=3572.7 (n=1195)**	**With de novo FIL PYE=1018.5 (n=335)**	**Total PYE=4591.2 (n=1530)**	**With continued FIL PYE=2568.5 (n=863)**	**With de novo FIL PYE=985.2 (n=336)**	**Total PYE=3553.8 (n=1199)**
Neoplasm malignant	1 (0.08)<br>0.0 (0.0–0.2)	-	1 (0.07)<br>0.0 (0.0–0.1)	-	-	-
Oesophageal squamous cell carcinoma metastatic	1 (0.08)<br>0.0 (0.0–0.2)	-	1 (0.07)<br>0.0 (0.0–0.1)	-	-	-
Osmotic demyelination syndrome	1 (0.08)<br>0.0 (0.0–0.2)	-	1 (0.07)<br>0.0 (0.0–0.1)	-	-	-
Ovarian cancer	-	-	-	1 (0.12)<br>0.0 (0.0–0.2)	-	1 (0.08)<br>0.0 (0.0–0.2)
Pericarditis	1 (0.08)<br>0.0 (0.0–0.2)	-	1 (0.07)<br>0.0 (0.0–0.1)	-	-	-
Pulmonary embolism	1 (0.08)<br>0.0 (0.0–0.2)	-	1 (0.07)<br>0.0 (0.0–0.1)	-	-	-
Renal cancer metastatic	-	1 (0.30)<br>0.1 (0.0–0.5)	1 (0.07)<br>0.0 (0.0–0.1)	-	-	-
Renal failure	-	-	-	1 (0.12)<br>0.0 (0.0–0.2)	-	1 (0.08)<br>0.0 (0.0–0.2)
Sepsis	-	-	-	-	1 (0.30)<br>0.1 (0.0–0.6)	1 (0.08)<br>0.0 (0.0–0.2)
Septic shock	1 (0.08)<br>0.0 (0.0–0.2)	-	1 (0.07)<br>0.0 (0.0–0.1)	-	-	-
Staphylococcal sepsis	1 (0.08)<br>0.0 (0.0–0.2)	-	1 (0.07)<br>0.0 (0.0–0.1)	-	-	-
Sudden death	-	-	-	1 (0.12)<br>0.0 (0.0–0.2)	-	1 (0.08)<br>0.0 (0.0–0.2)
Suspected COVID-19	-	-	-	-	1 (0.30)<br>0.1 (0.0–0.6)	1 (0.08)<br>0.0 (0.0–0.2)

TEAEs are defined as any adverse events that began on or after the study drug start date, up to 30 days after permanent discontinuation of study drug. Only adverse events with a start date after LTE treatment start are considered.

FIL(100/200), filgotinib (100 mg/200 mg); LTE, long-term extension; PYE, patient-years of exposure; TEAE, treatment-emergent adverse event.

Supplemental table 4 Incidence of TEAEs leading to premature discontinuation of filgotinib in FINCH 4

	FIL200 Number of patients (%) with the TEAE listed			FIL100 Number of patients (%) with the TEAE listed		
	**With continued FIL PYE=3572.7 (n=1195)**	**With de novo FIL PYE=1018.5 (n=335)**	**Total PYE=4591.2 (n=1530)**	**With continued FIL PYE=2568.5 (n=863)**	**With de novo FIL PYE=985.2 (n=336)**	**Total PYE=3553.8 (n=1199)**
COVID-19	20 (1.67)	2 (0.60)	22 (1.44)	5 (0.58)	5 (1.49)	10 (0.83)
COVID-19 pneumonia	8 (0.67)	2 (0.60)	10 (0.65)	4 (0.46)	4 (1.19)	8 (0.67)
Pneumonia	9 (0.75)	4 (1.19)	13 (0.85)	2 (0.23)	1 (0.30)	3 (0.25)
Hepatitis B DNA assay positive	2 (0.17)	3 (0.90)	5 (0.33)	4 (0.46)	2 (0.60)	6 (0.5)
Alanine aminotransferase increased	4 (0.33)	2 (0.60)	6 (0.39)	1 (0.12)	1 (0.30)	2 (0.17)
Pulmonary embolism	4 (0.33)	1 (0.30)	5 (0.33)	2 (0.23)		2 (0.17)
Latent tuberculosis	1 (0.08)	1 (0.30)	2 (0.13)	2 (0.23)	2 (0.60)	4 (0.33)
Rheumatoid arthritis	1 (0.08)	2 (0.60)	3 (0.20)	2 (0.23)	1 (0.30)	3 (0.25)
Aspartate aminotransferase increased	1 (0.08)	2 (0.60)	3 (0.20)	1 (0.12)	1 (0.30)	2 (0.17)
Creatinine renal clearance decreased	5 (0.42)		5 (0.33)			
Cellulitis	3 (0.25)		3 (0.20)	1 (0.12)		1 (0.08)
Pyelonephritis acute	2 (0.17)		2 (0.13)	1 (0.12)	1 (0.30)	2 (0.17)
Urinary tract infection	2 (0.17)		2 (0.13)	2 (0.23)		2 (0.17)
Acute kidney injury	2 (0.17)		2 (0.13)	1 (0.12)		1 (0.08)
Acute myocardial infarction	2 (0.17)		2 (0.13)	1 (0.12)		1 (0.08)
Adenocarcinoma of colon	1 (0.08)		1 (0.07)	2 (0.23)		2 (0.17)
Arthritis bacterial	2 (0.17)	1 (0.30)	3 (0.20)			
Gastric cancer	2 (0.17)		2 (0.13)	1 (0.12)		1 (0.08)
Hepatitis B virus test positive	2 (0.17)		2 (0.13)	1 (0.12)		1 (0.08)
Herpes zoster	2 (0.17)	1 (0.30)	3 (0.20)			

	FIL200 Number of patients (%) with the TEAE listed			FIL100 Number of patients (%) with the TEAE listed		
	**With continued FIL PYE=3572.7 (n=1195)**	**With de novo FIL PYE=1018.5 (n=335)**	**Total PYE=4591.2 (n=1530)**	**With continued FIL PYE=2568.5 (n=863)**	**With de novo FIL PYE=985.2 (n=336)**	**Total PYE=3553.8 (n=1199)**
Malignant melanoma	3 (0.25)		3 (0.20)			
Mycobacterium tuberculosis complex test positive	1 (0.08)		1 (0.07)	1 (0.12)	1 (0.30)	2 (0.17)
Neutropenia				1 (0.12)	2 (0.60)	3 (0.25)
Osteomyelitis	2 (0.17)		2 (0.13)	1 (0.12)		1 (0.08)
Prostate cancer	1 (0.08)	1 (0.30)	2 (0.13)		1 (0.30)	1 (0.08)
Renal failure	1 (0.08)	1 (0.30)	2 (0.13)	1 (0.12)		1 (0.08)
Uterine cancer				1 (0.12)	2 (0.60)	3 (0.25)
Abdominal pain				2 (0.23)		2 (0.17)
Acute respiratory failure	1 (0.08)		1 (0.07)	1 (0.12)		1 (0.08)
Anaemia	1 (0.08)		1 (0.07)	1 (0.12)		1 (0.08)
Chronic kidney disease	1 (0.08)		1 (0.07)	1 (0.12)		1 (0.08)
Deep vein thrombosis	1 (0.08)		1 (0.07)	1 (0.12)		1 (0.08)
Diabetic ketoacidosis	1 (0.08)		1 (0.07)		1 (0.30)	1 (0.08)
Diffuse large B-cell lymphoma	2 (0.17)		2 (0.13)			
Diverticulitis	1 (0.08)		1 (0.07)	1 (0.12)		1 (0.08)
Endometrial cancer				2 (0.23)		2 (0.17)
Gamma-glutamyltransferase increased	2 (0.17)		2 (0.13)			
Hepatic cirrhosis		1 (0.30)	1 (0.07)	1 (0.12)		1 (0.08)
Hepatitis B		1 (0.30)	1 (0.70)	1 (0.12)		1 (0.08)
Hypertension	2 (0.17)		2 (0.13)			
Hypertransaminasaemia	1 (0.08)		1 (0.07)	1 (0.12)		1 (0.08)
Hypokalaemia	2 (0.17)		2 (0.13)			

	FIL200<br>Number of patients (%) with the TEAE listed			FIL100<br>Number of patients (%) with the TEAE listed		
	**With continued FIL PYE=3572.7 (n=1195)**	**With de novo FIL PYE=1018.5 (n=335)**	**Total PYE=4591.2 (n=1530)**	**With continued FIL PYE=2568.5 (n=863)**	**With de novo FIL PYE=985.2 (n=336)**	**Total PYE=3553.8 (n=1199)**
Infectious pleural effusion	1 (0.08)	1 (0.30)	2 (0.13)			
Interstitial lung disease		1 (0.30)	1 (0.07)	1 (0.12)		1 (0.08)
Leukopenia				1 (0.12)	1 (0.30)	2 (0.17)
Lymphocyte count decreased		1 (0.30)	1 (0.07)	1 (0.12)		1 (0.08)
Pleural effusion	1 (0.08)		1 (0.07)	1 (0.12)		1 (0.08)
Postoperative wound infection	1 (0.08)		1 (0.07)	1 (0.12)		1 (0.08)
Pyelonephritis				1 (0.12)	1 (0.30)	2 (0.17)
Pyonephrosis		1 (0.30)	1 (0.07)	1 (0.12)		1 (0.08)
Respiratory failure	1 (0.08)		1 (0.07)	1 (0.12)		1 (0.08)
Sepsis	1 (0.08)		1 (0.07)		1 (0.30)	1 (0.08)
Staphylococcal sepsis	1 (0.08)		1 (0.07)	1 (0.12)		1 (0.08)
Thrombocytopenia	2 (0.17)		2 (0.13)			
Urosepsis				2 (0.23)		2 (0.17)
Abscess limb	1 (0.08)		1 (0.07)			
Acute respiratory distress syndrome	1 (0.08)		1 (0.07)			
Acute sinusitis	1 (0.08)		1 (0.07)			
Adenocarcinoma	1 (0.08)		1 (0.07)			
Adenocarcinoma gastric		1 (0.03)	1 (0.07)			
Adenocarcinoma of appendix					1 (0.30)	1 (0.08)
Adenocarcinoma of the cervix				1 (0.12)		1 (0.08)
Adenocarcinoma pancreas				1 (0.12)		1 (0.08)

	FIL200 Number of patients (%) with the TEAE listed			FIL100 Number of patients (%) with the TEAE listed		
	With continued FIL PYE=3572.7 (n=1195)	With de novo FIL PYE=1018.5 (n=335)	Total PYE=4591.2 (n=1530)	With continued FIL PYE=2568.5 (n=863)	With de novo FIL PYE=985.2 (n=336)	Total PYE=3553.8 (n=1199)
Adenomyosis	1 (0.08)		1 (0.07)			
Anxiety		1 (0.30)	1 (0.07)			
Aortic aneurysm	1 (0.08)		1 (0.07)			
Arthralgia		1 (0.30)	1 (0.07)			
Ascites				1 (0.12)		1 (0.08)
Atrial fibrillation	1 (0.08)		1 (0.07)			
Atypical pneumonia				1 (0.12)		1 (0.08)
B-cell lymphoma		1 (0.30)	1 (0.07)			
Bacteraemia	1 (0.08)		1 (0.07)			
Basal cell carcinoma				1 (0.12)		1 (0.08)
Bile duct cancer		1 (0.30)	1 (0.07)			
Bladder cancer		1 (0.30)	1 (0.07)			
Bladder transitional cell carcinoma				1 (0.12)		1 (0.08)
Blood creatine phosphokinase increased					1 (0.30)	1 (0.08)
Brain oedema	1 (0.08)		1 (0.07)			
Breast cancer	1 (0.08)		1 (0.07)			
Brucellosis	1 (0.08)		1 (0.07)			
Candida pneumonia					1 (0.30)	1 (0.08)
Carcinoid tumour					1 (0.30)	1 (0.08)
Cardiac arrest	1 (0.08)		1 (0.07)			
Central nervous system lymphoma				1 (0.12)		1 (0.08)
Cerebral small vessel ischaemic disease				1 (0.12)		1 (0.08)

	FIL200 Number of patients (%) with the TEAE listed			FIL100 Number of patients (%) with the TEAE listed		
	**With continued FIL PYE=3572.7 (n=1195)**	**With de novo FIL PYE=1018.5 (n=335)**	**Total PYE=4591.2 (n=1530)**	**With continued FIL PYE=2568.5 (n=863)**	**With de novo FIL PYE=985.2 (n=336)**	**Total PYE=3553.8 (n=1199)**
Cerebrovascular accident					1 (0.30)	1 (0.08)
Cervix carcinoma	1 (0.08)		1 (0.07)			
Chikungunya virus infection	1 (0.08)		1 (0.07)			
Cholecystitis acute	1 (0.08)		1 (0.07)			
Chronic obstructive pulmonary disease	1 (0.08)		1 (0.07)			
Community acquired infection	1 (0.08)		1 (0.07)			
Conjunctival melanoma	1 (0.08)		1 (0.07)			
Deafness neurosensory	1 (0.08)		1 (0.07)			
Dehydration	1 (0.08)		1 (0.07)			
Dementia				1 (0.12)		1 (0.08)
Device dislocation				1 (0.12)		1 (0.08)
Diverticulum intestinal				1 (0.12)		1 (0.08)
Diverticulum intestinal haemorrhagic				1 (0.12)		1 (0.08)
Dizziness		1 (0.30)	1 (0.07)			
Drug-induced liver injury	1 (0.08)		1 (0.07)			
Dyspnoea		1 (0.30)	1 (0.07)			
Encephalopathy	1 (0.08)		1 (0.07)			
Endometrial adenocarcinoma					1 (0.30)	1 (0.08)
Endometrial hyperplasia	1 (0.08)		1 (0.07)			
Epstein-Barr viraemia	1 (0.08)		1 (0.07)			
Erysipelas	1 (0.08)		1 (0.07)			
Extradural abscess	1 (0.08)		1 (0.07)			

	FIL200<br>Number of patients (%) with the TEAE listed			FIL100<br>Number of patients (%) with the TEAE listed		
	**With continued FIL PYE=3572.7 (n=1195)**	**With de novo FIL PYE=1018.5 (n=335)**	**Total PYE=4591.2 (n=1530)**	**With continued FIL PYE=2568.5 (n=863)**	**With de novo FIL PYE=985.2 (n=336)**	**Total PYE=3553.8 (n=1199)**
Femur fracture				1 (0.12)		1 (0.08)
Flank pain		1 (0.30)	1 (0.07)			
Gastric perforation	1 (0.08)		1 (0.07)			
Gastric ulcer perforation				1 (0.12)		1 (0.08)
Gastroenteritis				1 (0.12)		1 (0.08)
Gastroenteritis salmonella				1 (0.12)		1 (0.08)
Gastrointestinal perforation				1 (0.12)		1 (0.08)
Gastrointestinal stromal tumour	1 (0.08)		1 (0.07)			
H1N1 influenza				1 (0.12)		1 (0.08)
Haemoptysis				1 (0.12)		1 (0.08)
Hepatic steatosis				1 (0.12)		1 (0.08)
Herpes zoster cutaneous disseminated	1 (0.08)		1 (0.07)			
Herpes zoster disseminated	1 (0.08)		1 (0.07)			
Hilar lymphadenopathy	1 (0.08)		1 (0.07)			
Human ehrlichiosis	1 (0.08)		1 (0.07)			
Hydrometra				1 (0.12)		1 (0.08)
Hypertensive crisis	1 (0.08)		1 (0.07)			
Hypertonic bladder				1 (0.12)		1 (0.08)
Hypomagnesaemia	1 (0.08)		1 (0.07)			
Hyponatraemia	1 (0.08)		1 (0.07)			
Infected skin ulcer	1 (0.08)		1 (0.07)			
Infection	1 (0.08)		1 (0.07)			
Infective keratitis	1 (0.08)		1 (0.07)			

	FIL200<br>Number of patients (%) with the TEAE listed			FIL100<br>Number of patients (%) with the TEAE listed		
	**With continued FIL<br>PYE=3572.7<br>(n=1195)**	**With de novo FIL<br>PYE=1018.5<br>(n=335)**	**Total<br>PYE=4591.2<br>(n=1530)**	**With continued FIL<br>PYE=2568.5<br>(n=863)**	**With de novo FIL<br>PYE=985.2<br>(n=336)**	**Total<br>PYE=3553.8<br>(n=1199)**
Inflammation				1 (0.12)		1 (0.08)
Intraductal proliferative breast lesion	1 (0.08)		1 (0.07)			
Invasive breast carcinoma	1 (0.08)		1 (0.07)			
Ischaemic stroke	1 (0.08)		1 (0.07)			
Liver disorder				1 (0.12)		1 (0.08)
Localised infection	1 (0.08)		1 (0.07)			
Lung adenocarcinoma	1 (0.08)		1 (0.07)			
Lung cancer metastatic	1 (0.08)		1 (0.07)			
Lung disorder				1 (0.12)		1 (0.08)
Lung neoplasm				1 (0.12)		1 (0.08)
Lymph node tuberculosis				1 (0.12)		1 (0.08)
Malignant neoplasm of unknown primary site		1 (0.30)	1 (0.07)			
Malignant peritoneal neoplasm				1 (0.12)		1 (0.08)
Mental impairment				1 (0.12)		1 (0.08)
Metastases to bone		1 (0.30)	1 (0.07)			
Metastases to liver		1 (0.30)	1 (0.07)			
Metastases to salivary gland		1 (0.30)	1 (0.07)			
Metastatic neoplasm				1 (0.12)		1 (0.08)
Microscopic polyangiitis	1 (0.08)		1 (0.07)			
Mouth ulceration	1 (0.08)		1 (0.07)			
Myocardial infarction				1 (0.12)		1 (0.08)
Nasal sinus cancer				1 (0.12)		1 (0.08)

	FIL200 Number of patients (%) with the TEAE listed			FIL100 Number of patients (%) with the TEAE listed		
	**With continued FIL PYE=3572.7 (n=1195)**	**With de novo FIL PYE=1018.5 (n=335)**	**Total PYE=4591.2 (n=1530)**	**With continued FIL PYE=2568.5 (n=863)**	**With de novo FIL PYE=985.2 (n=336)**	**Total PYE=3553.8 (n=1199)**
Necrotising fasciitis	1 (0.08)		1 (0.07)			
Nephrolithiasis		1 (0.30)	1 (0.07)			
Neuroendocrine carcinoma of the skin	1 (0.08)		1 (0.07)			
Non-Hodgkin's lymphoma				1 (0.12)		1 (0.08)
Non-small cell lung cancer		1 (0.30)	1 (0.07)			
Not coded				1 (0.12)		1 (0.08)
Oesophageal candidiasis	1 (0.08)		1 (0.07)			
Oesophageal carcinoma				1 (0.12)		1 (0.08)
Oesophageal squamous cell carcinoma metastatic	1 (0.08)		1 (0.07)			
Osmotic demyelination syndrome	1 (0.08)		1 (0.07)			
Otitis media acute	1 (0.08)		1 (0.07)			
Ovarian cancer				1 (0.12)		1 (0.08)
Ovarian granulosa cell tumour	1 (0.08)		1 (0.07)			
Ovarian neoplasm					1 (0.30)	1 (0.08)
Pancreatitis acute				1 (0.12)		1 (0.08)
Pancreatitis chronic				1 (0.12)		1 (0.08)
Pancytopenia				1 (0.12)		1 (0.08)
Papillary thyroid cancer	1 (0.08)		1 (0.07)			
Paraplegia					1 (0.30)	1 (0.08)
Paraspinal abscess	1 (0.08)		1 (0.07)			
Peripheral artery aneurysm	1 (0.08)		1 (0.07)			

	FIL200<br>Number of patients (%) with the TEAE listed			FIL100<br>Number of patients (%) with the TEAE listed		
	**With continued FIL<br>PYE=3572.7<br>(n=1195)**	**With de novo FIL<br>PYE=1018.5<br>(n=335)**	**Total<br>PYE=4591.2<br>(n=1530)**	**With continued FIL<br>PYE=2568.5<br>(n=863)**	**With de novo FIL<br>PYE=985.2<br>(n=336)**	**Total<br>PYE=3553.8<br>(n=1199)**
Pharyngeal cancer metastatic	1 (0.08)		1 (0.07)			
Plasma cell myeloma				1 (0.12)		1 (0.08)
Pleuritic pain	1 (0.08)		1 (0.07)			
Pneumonia aspiration	1 (0.08)		1 (0.07)			
Pneumonia cryptococcal	1 (0.08)		1 (0.07)			
Pneumonia pneumococcal	1 (0.08)		1 (0.07)			
Pneumonia viral		1 (0.30)	1 (0.07)			
Pneumonitis		1 (0.30)	1 (0.07)			
Post procedural infection				1 (0.12)		1 (0.08)
Prostate cancer metastatic		1 (0.30)	1 (0.07)			
Pulmonary mass				1 (0.12)		1 (0.08)
Pulmonary oedema	1 (0.08)		1 (0.07)			
Pulmonary tuberculosis	1 (0.08)		1 (0.07)			
Pyelonephritis chronic	1 (0.08)		1 (0.07)			
Rectal neoplasm				1 (0.12)		1 (0.08)
Rectosigmoid cancer	1 (0.08)		1 (0.07)			
Renal cancer	1 (0.08)		1 (0.07)			
Renal cancer metastatic		1 (0.30)	1 (0.07)			
Renal cell carcinoma				1 (0.12)		1 (0.08)
Renal function test abnormal				1 (0.12)		1 (0.08)
Renal impairment				1 (0.12)		1 (0.08)
Restlessness				1 (0.12)		1 (0.08)
SARS-CoV-2 test positive					1 (0.30)	1 (0.08)

	FIL200 Number of patients (%) with the TEAE listed			FIL100 Number of patients (%) with the TEAE listed		
	**With continued FIL PYE=3572.7 (n=1195)**	**With de novo FIL PYE=1018.5 (n=335)**	**Total PYE=4591.2 (n=1530)**	**With continued FIL PYE=2568.5 (n=863)**	**With de novo FIL PYE=985.2 (n=336)**	**Total PYE=3553.8 (n=1199)**
Seborrhoeic dermatitis	1 (0.08)		1 (0.07)			
Sinus arrhythmia					1 (0.30)	1 (0.08)
Sinus bradycardia					1 (0.30)	1 (0.08)
Sinus tachycardia	1 (0.08)		1 (0.07)			
Skin ulcer	1 (0.08)		1 (0.07)			
Spinal osteoarthritis				1 (0.12)		1 (0.08)
Squamous cell carcinoma of the skin	1 (0.08)		1 (0.07)			
Staphylococcal bacteraemia				1 (0.12)		1 (0.08)
Stroma site infection				1 (0.12)		1 (0.08)
Stress cardiomyopathy	1 (0.08)		1 (0.07)			
Subarachnoid haemorrhage					1 (0.30)	1 (0.08)
Subcutaneous emphysema	1 (0.08)		1 (0.07)			
Superficial vein thrombosis	1 (0.08)		1 (0.07)			
Supraventricular tachycardia				1 (0.12)		1 (0.08)
Syphilis				1 (0.12)		1 (0.08)
T-cell lymphoma					1 (0.30)	1 (0.08)
Thrombotic cerebral infarction	1 (0.08)		1 (0.07)			
Thyroid mass	1 (0.08)		1 (0.07)			
Tibia fracture				1 (0.12)		1 (0.08)
Transitional cell carcinoma					1 (0.30)	1 (0.08)
Tremor	1 (0.08)		1 (0.07)			
Tuberculosis				1 (0.12)		1 (0.08)

	FIL200<br>Number of patients (%) with the TEAE listed			FIL100<br>Number of patients (%) with the TEAE listed		
	**With continued FIL PYE=3572.7 (n=1195)**	**With de novo FIL PYE=1018.5 (n=335)**	**Total PYE=4591.2 (n=1530)**	**With continued FIL PYE=2568.5 (n=863)**	**With de novo FIL PYE=985.2 (n=336)**	**Total PYE=3553.8 (n=1199)**
Urinary tract infection bacterial				1 (0.12)		1 (0.08)
Urticarial vasculitis				1 (0.12)		1 (0.08)
Vaginal haemorrhage				1 (0.12)		1 (0.08)
Varicella	1 (0.08)		1 (0.07)			
Vomiting	1 (0.08)		1 (0.07)			
Wound infection					1 (0.30)	1 (0.08)

TEAEs are defined as any adverse events that began on or after the study drug start date, up to 30 days after permanent discontinuation of study drug. Only adverse events with a start date after LTE treatment start are considered.

FIL(100/200), filgotinib (100 mg/200 mg); LTE, long-term extension; PYE, patient-years of exposure; TEAE, treatment-emergent adverse event.

Supplemental figure 1 Study design

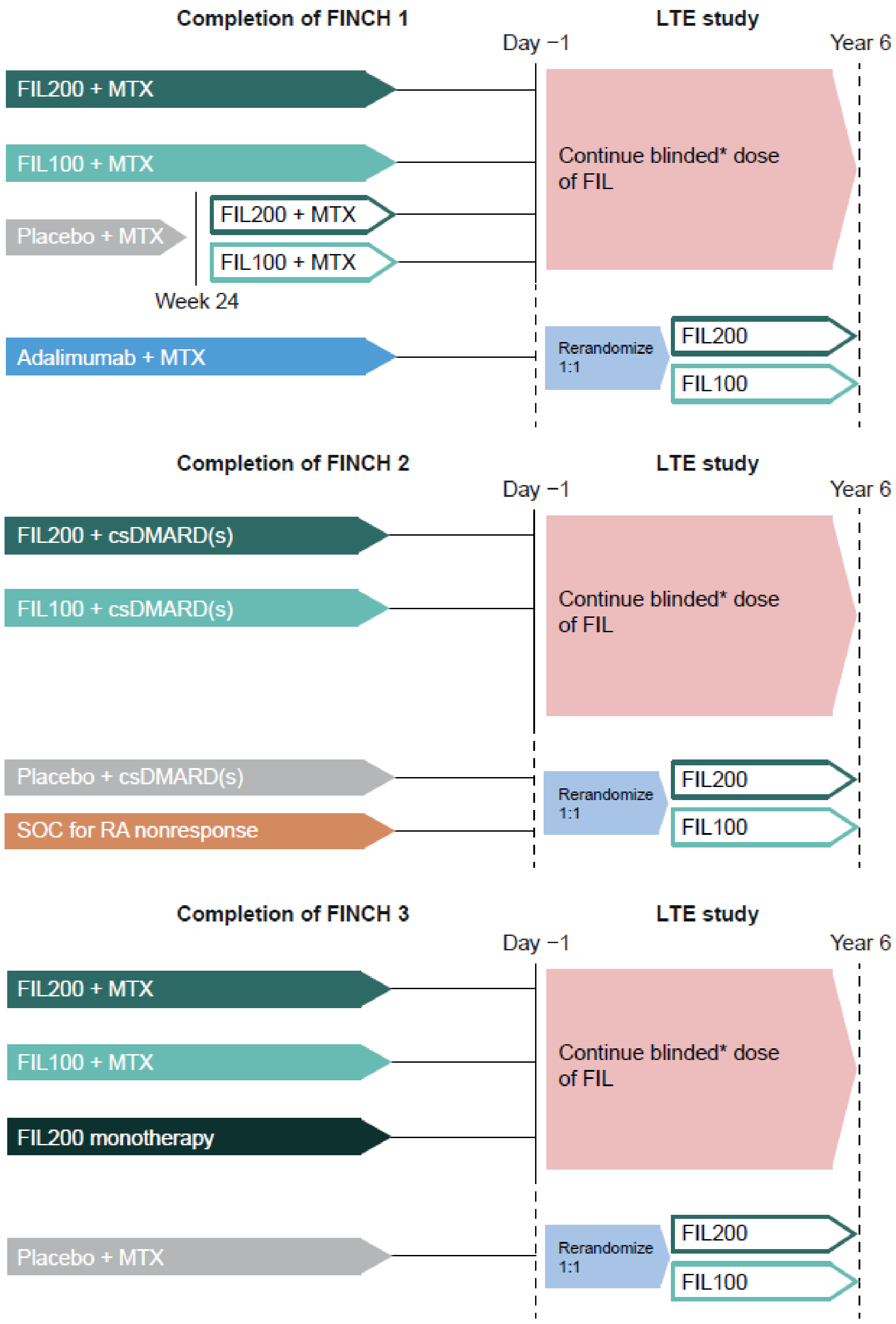

*Until study becomes open label.

csDMARD, conventional synthetic disease-modifying anti-rheumatic drug; FIL(100/200), filgotinib (100 mg/200 mg); LTE, long-term extension; MTX, methotrexate; RA, rheumatoid arthritis; SOC, standard of care.

Supplemental figure 2 The proportion of patients who achieved ACR20 in FINCH 4 according to parent study: FINCH 1 (A), FINCH 2 (B) and FINCH 3 (C) (safety analysis set, OC)

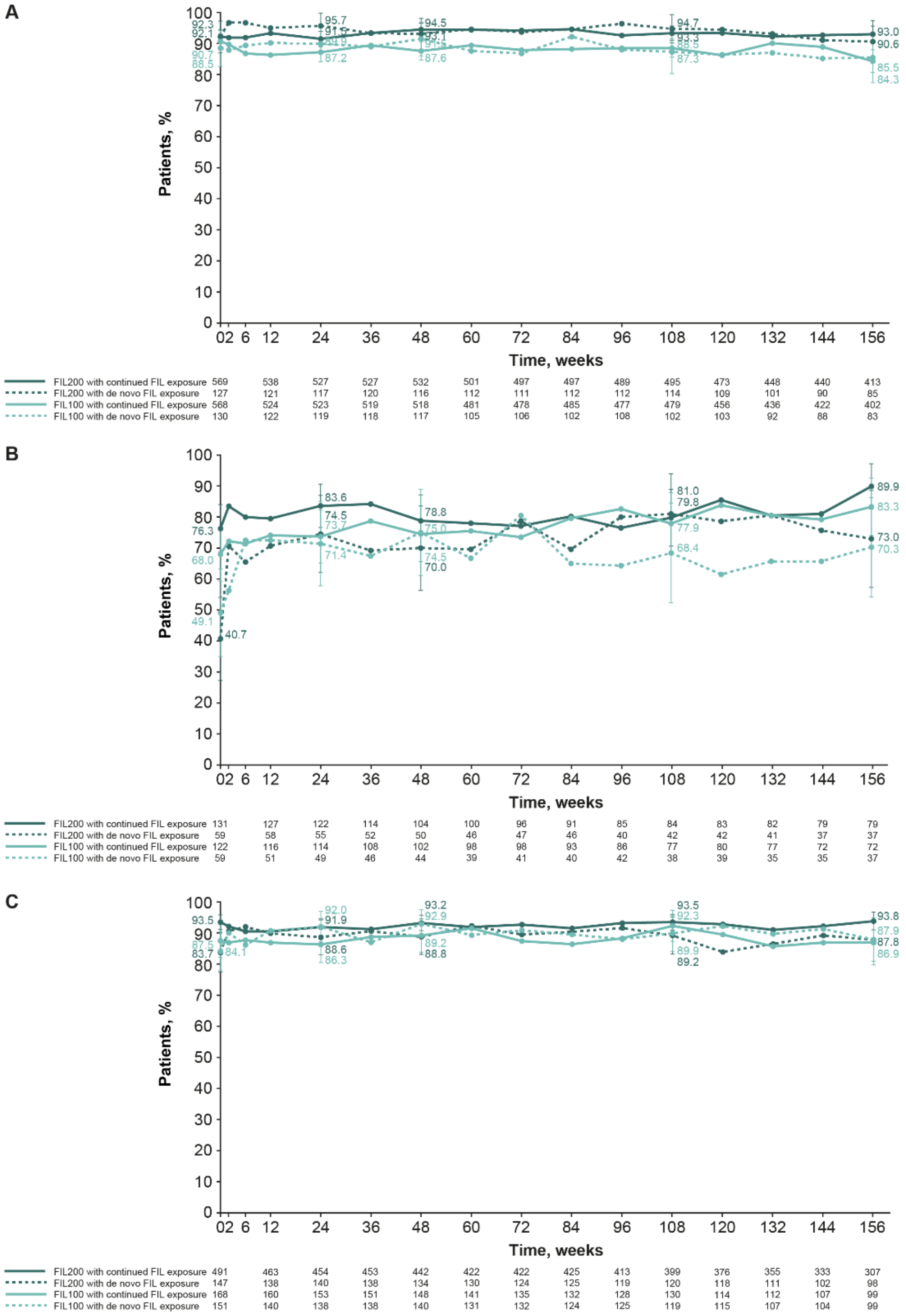

ACR20 was calculated based on parent study baseline. Error bars show 95% CIs. ACR20, American College of Rheumatology 20% response; FIL(100/200), filgotinib (100 mg/200 mg); OC, observed case.

Supplemental figure 3 The proportion of patients who achieved ACR50 in FINCH 4 according to parent study: FINCH 1 (A), FINCH 2 (B) and FINCH 3 (C) (safety analysis set, NRI)

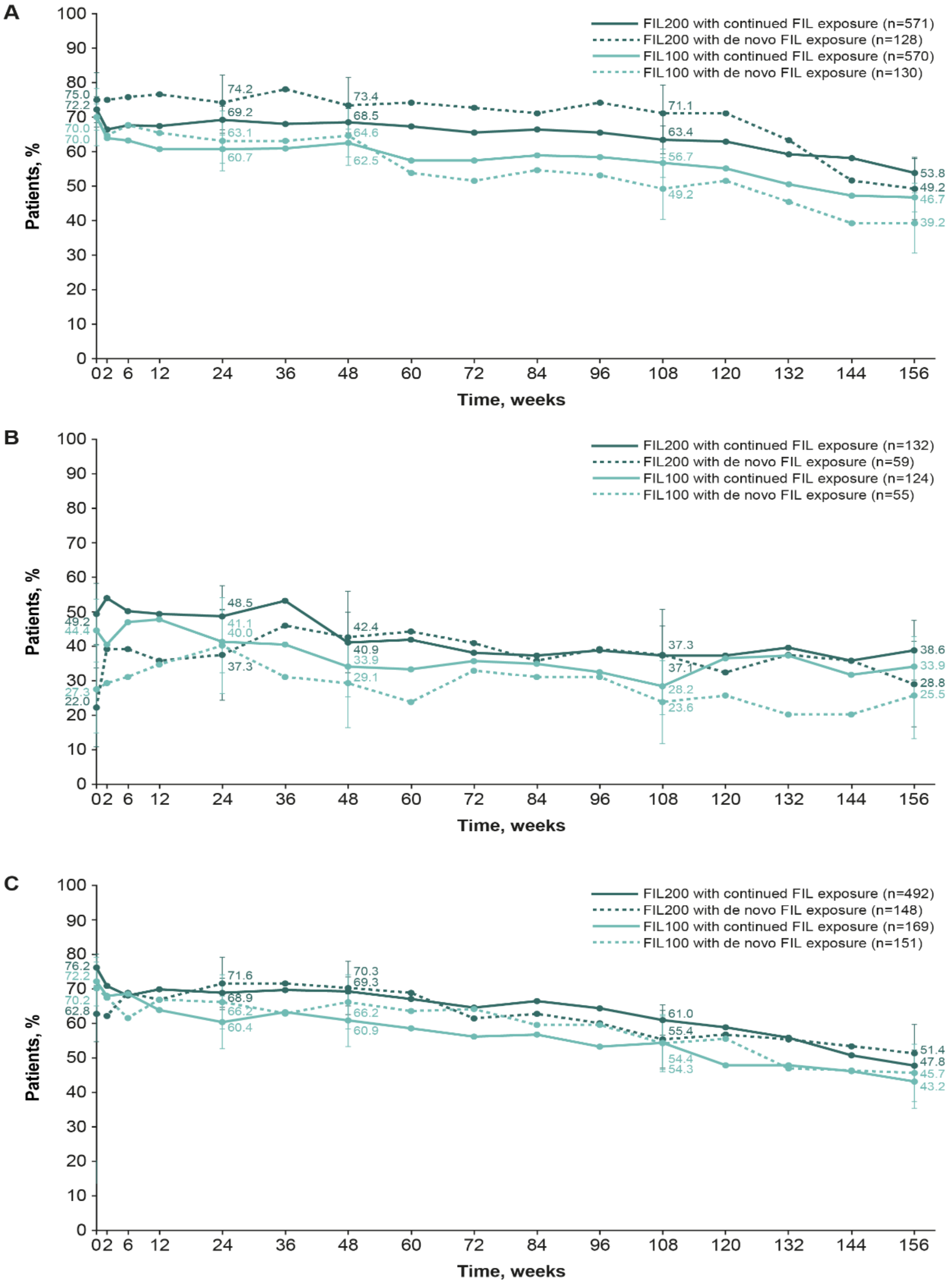

Patients with missing outcomes were set as non-responders. ACR50 was calculated based on the parent study baseline. Error bars show 95% CIs.

ACR50, American College of Rheumatology 50% response; FIL(100/200), filgotinib (100 mg/200 mg); NRI, non-responder imputation.

Supplemental figure 4 The proportion of patients who achieved ACR70 responses in FINCH 4 according to parent study: FINCH 1 (A), FINCH 2 (B) and FINCH 3 (C) (safety analysis set, NRI)

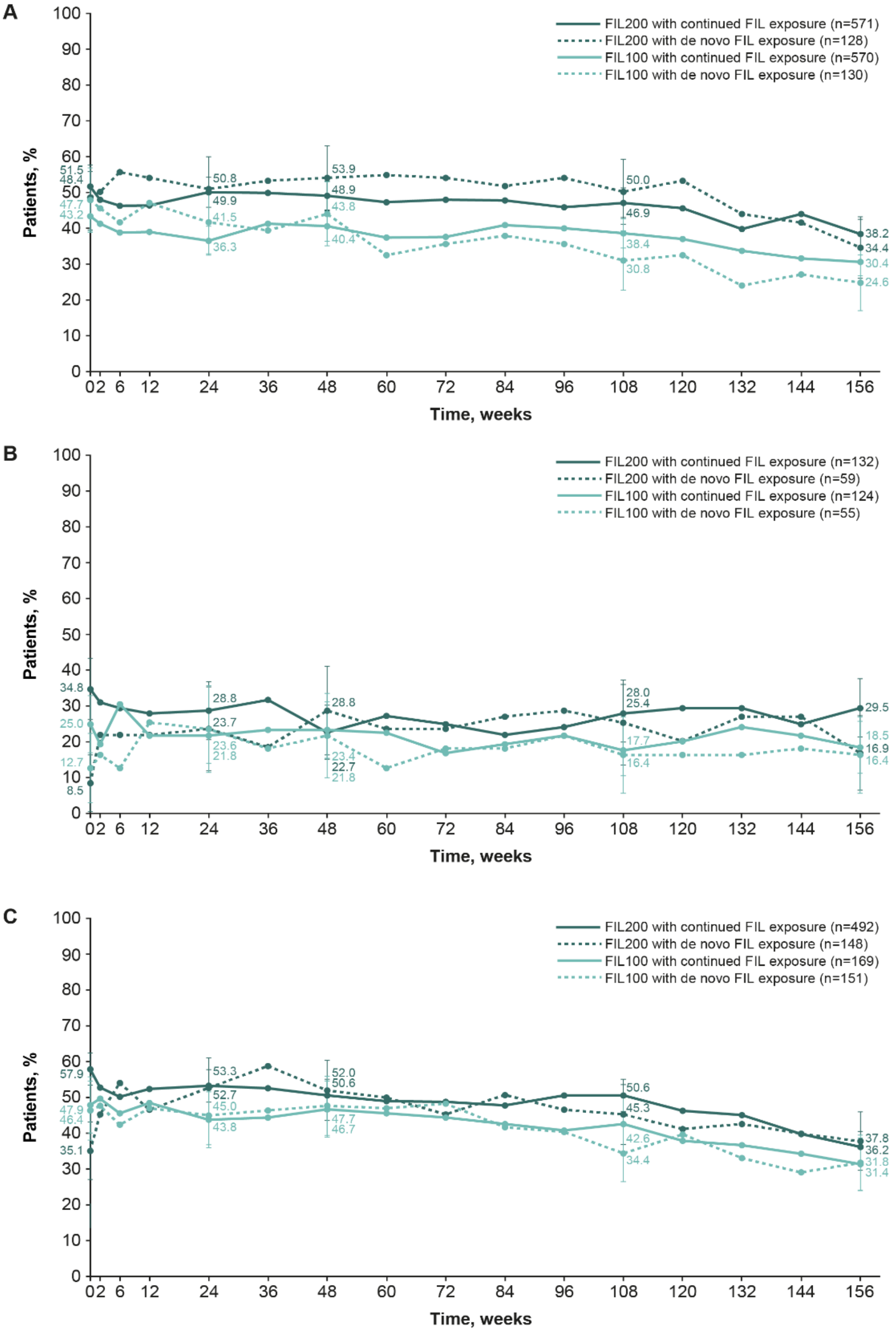

Patients with missing outcomes were set as non-responders. ACR70 was calculated based on the parent study baseline. Error bars show 95% CIs.

ACR70, American College of Rheumatology 70% response; FIL(100/200), filgotinib (100 mg/200 mg); NRI, non-responder imputation.

Supplemental figure 5 The proportion of patients who achieved ACR50 in FINCH 4 according to parent study: FINCH 1 (A), FINCH 2 (B) and FINCH 3 (C) (safety analysis set, OC)

A

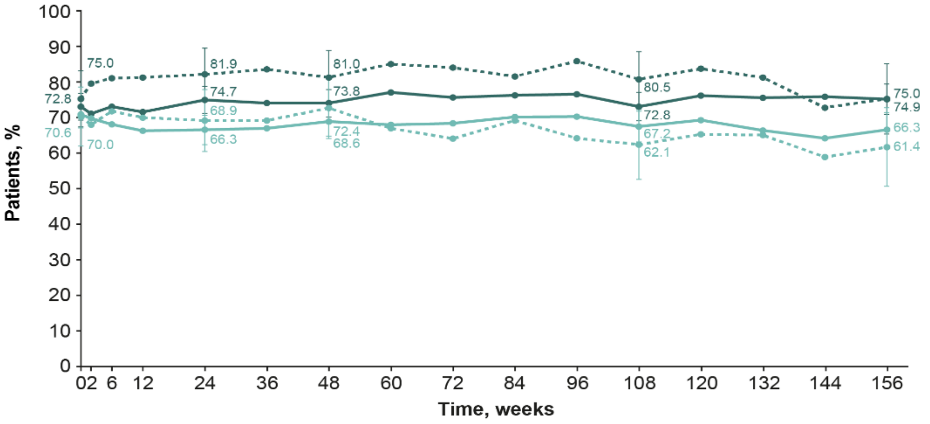

FIL200 with continued FIL exposure	566	540	529	526	530	500	496	499	490	497	473	449	439	410
FIL200 with de novo FIL exposure	128	121	116	120	116	112	111	112	111	113	109	100	91	84
FIL100 with continued FIL exposure	565	524	522	520	519	483	480	481	476	481	455	436	421	401
FIL100 with de novo FIL exposure	130	122	119	119	116	105	105	103	108	103	103	91	87	83

B

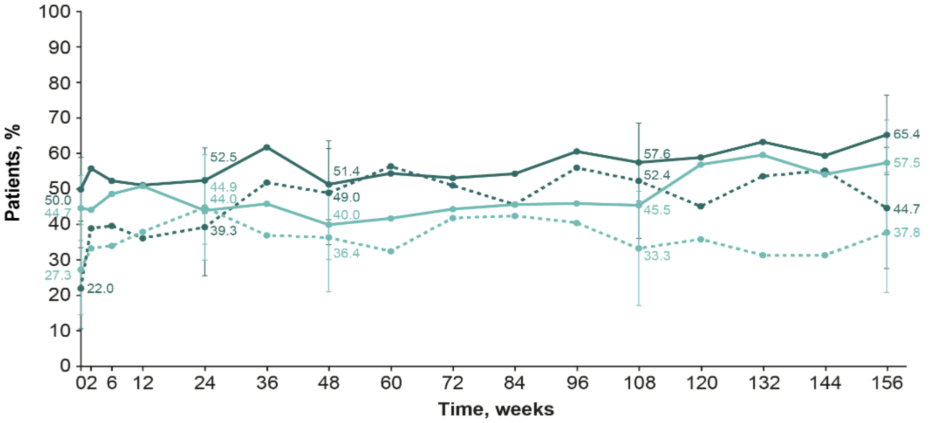

FIL200 with continued FIL exposure	130	127	122	113	105	101	94	90	84	85	83	82	79	78
FIL200 with de novo FIL exposure	59	58	56	52	51	46	47	46	41	42	42	41	38	38
FIL100 with continued FIL exposure	123	116	116	109	105	98	99	94	87	77	79	77	72	73
FIL100 with de novo FIL exposure	55	50	49	46	44	40	43	40	42	39	39	35	35	37

C

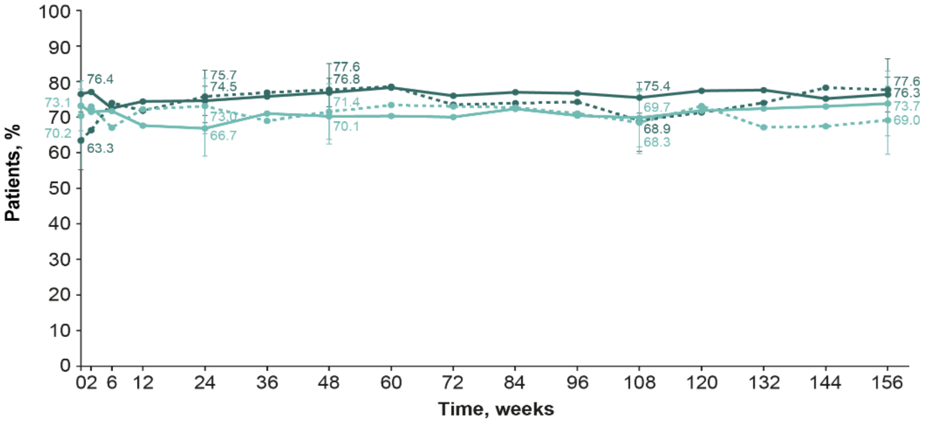

FIL200 with continued FIL exposure	491	463	455	453	444	422	419	425	414	398	375	355	333	308
FIL200 with de novo FIL exposure	147	138	140	138	134	130	124	126	120	119	118	111	101	98
FIL100 with continued FIL exposure	167	160	153	151	147	141	136	133	128	132	113	112	107	99
FIL100 with de novo FIL exposure	151	140	137	138	140	131	133	124	127	120	115	106	104	100

ACR50 was calculated based on the parent study baseline. Error bars show 95% CIs. ACR50, American College of Rheumatology 50% response; FIL(100/200), filgotinib (100 mg/200 mg); OC, observed case.

Supplemental figure 6 The proportion of patients who achieved ACR70 in FINCH 4 according to parent study: FINCH 1 (A), FINCH 2 (B) and FINCH 3 (C) (safety analysis set, OC)

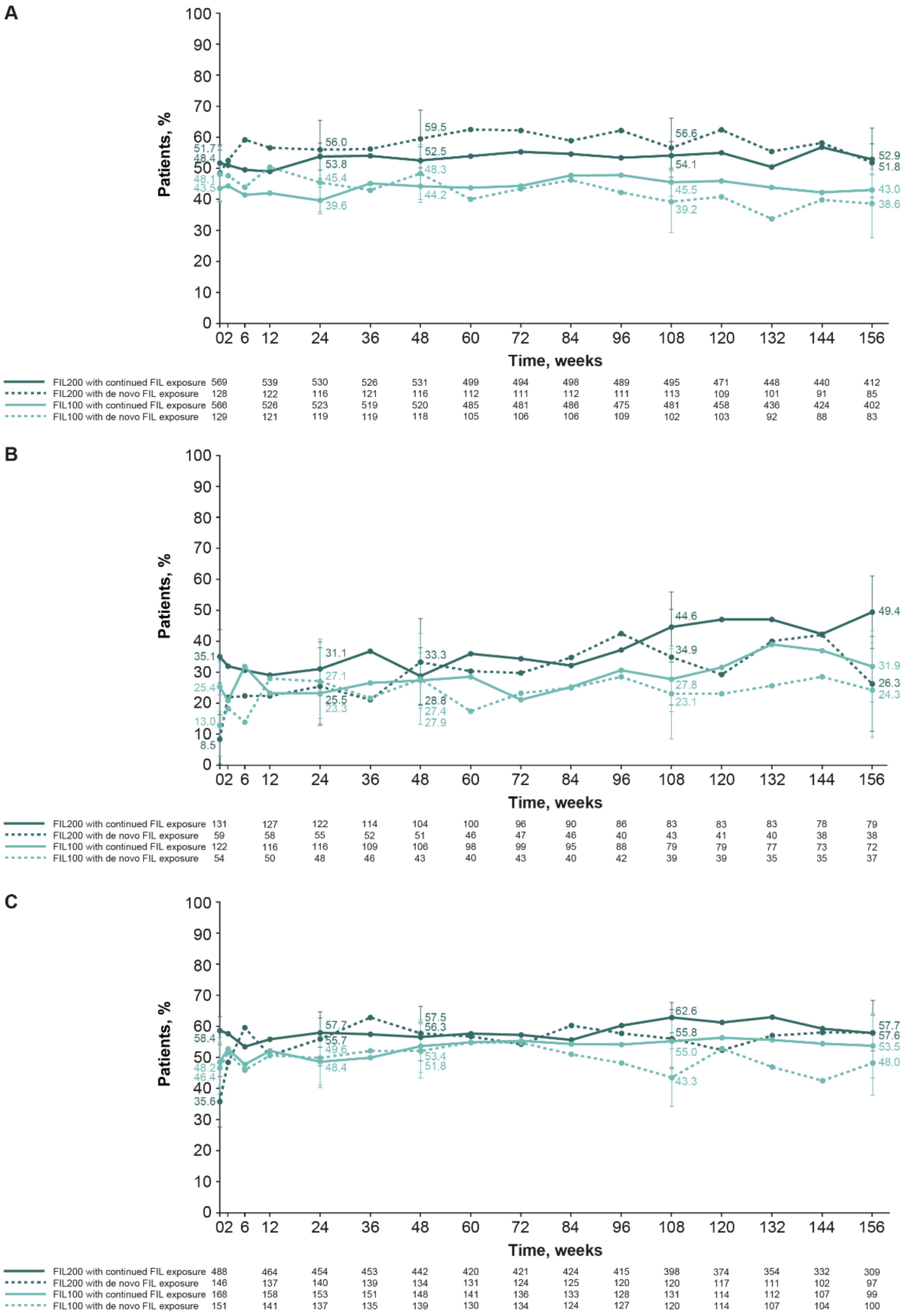

ACR70 was calculated based on the parent study baseline. Error bars show 95% CIs. ACR70, American College of Rheumatology 70% response; FIL(100/200), filgotinib (100 mg/200 mg); OC, observed case.

Supplemental figure 7 The proportion of patients who achieved DAS28-CRP <2.6 in FINCH 4 according to parent study: FINCH 1 (A), FINCH 2 (B) and FINCH 3 (C) (safety analysis set, OC)

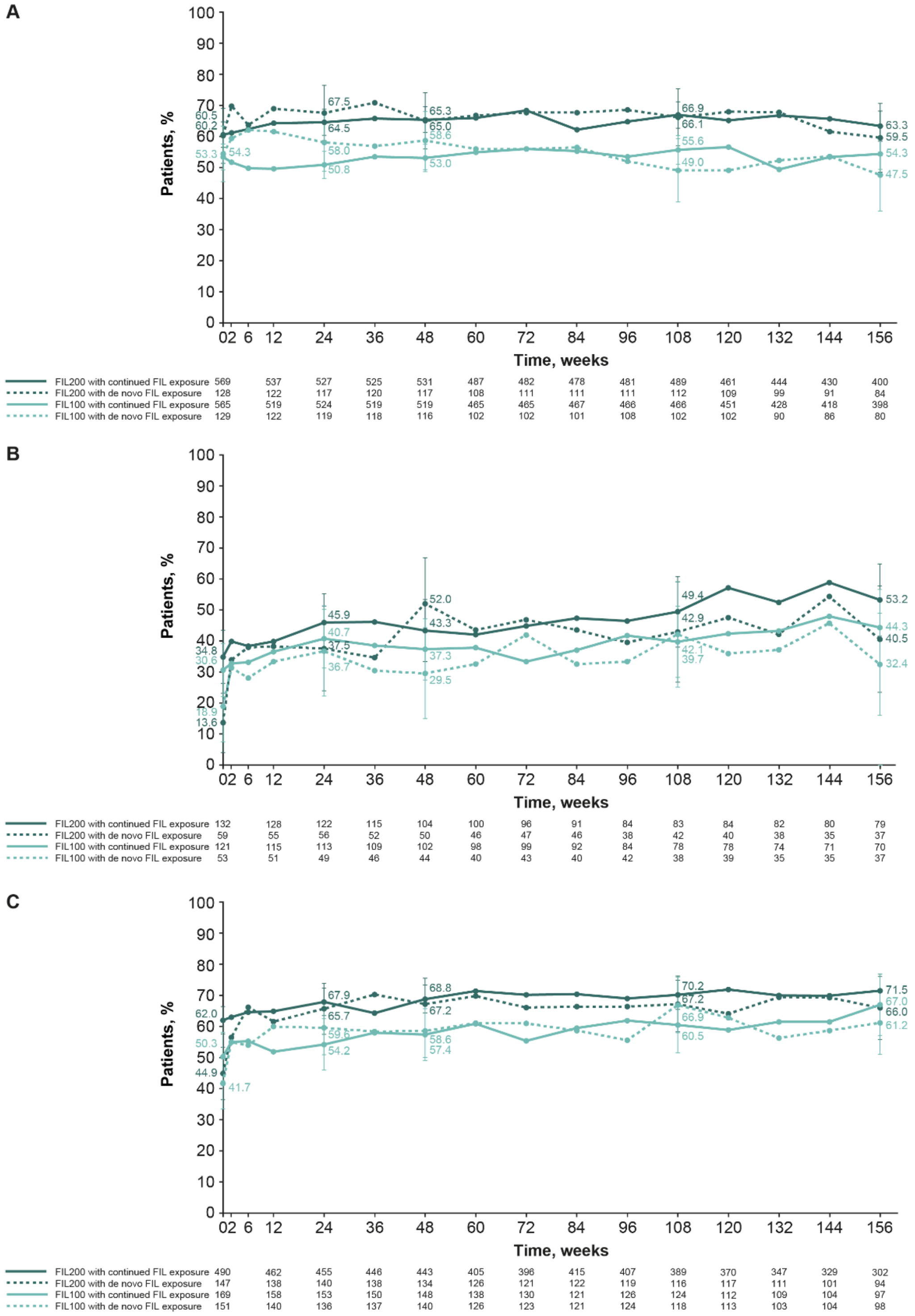

DAS28-CRP was calculated based on the parent study baseline. Error bars show 95% CIs. DAS28-CRP, Disease Activity Score 28 using C-reactive protein; FIL(100/200), filgotinib (100 mg/200 mg); OC, observed case.

Supplemental figure 8 The proportion of patients who achieved SDAI ≤3.3 in FINCH 4 according to parent study: FINCH 1 (A), FINCH 2 (B) and FINCH 3 (C) (safety analysis set, NRI)

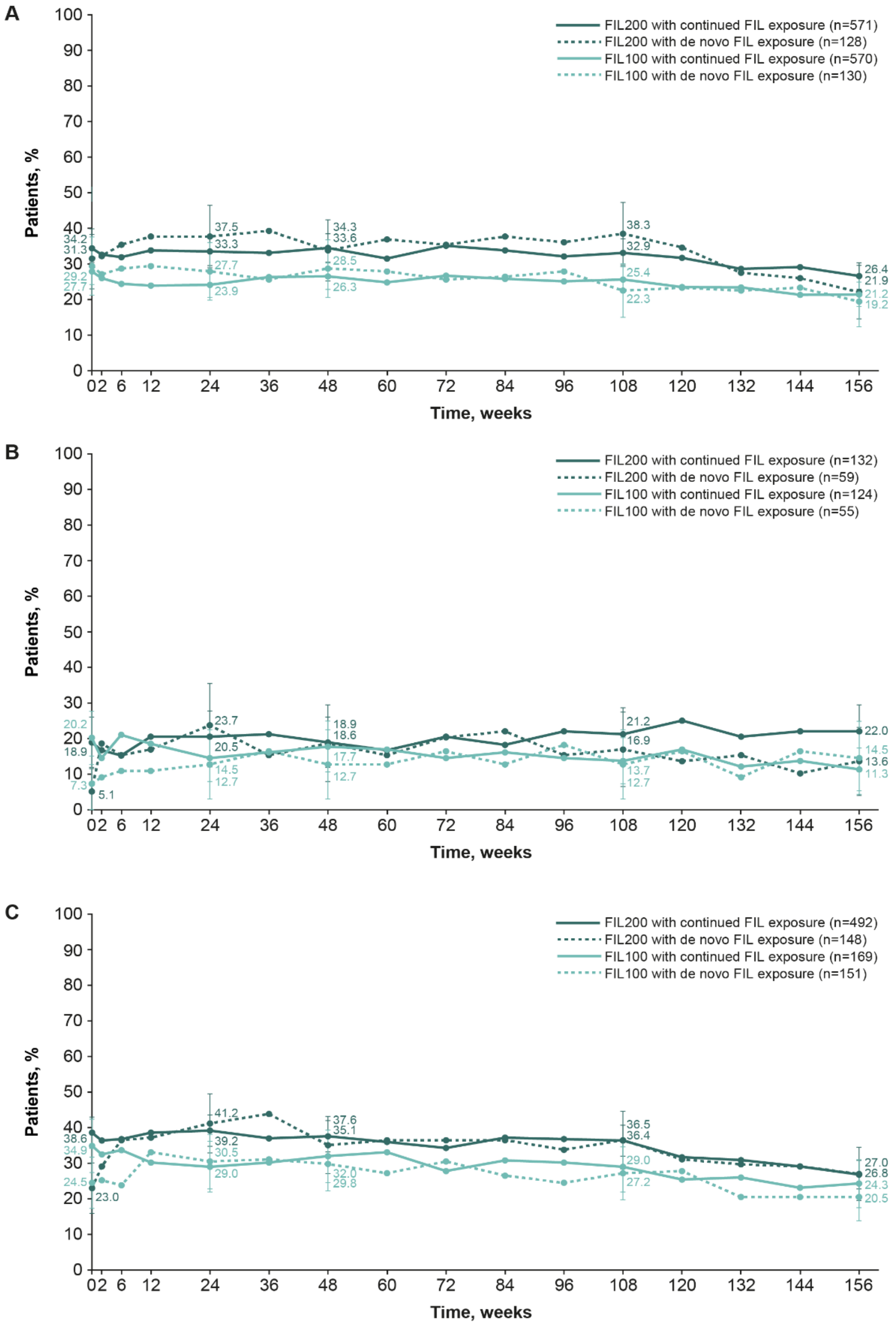

Patients with missing outcomes were set as non-responders. Error bars show 95% CIs. FIL(100/200), filgotinib (100 mg/200 mg); NRI, non-responder imputation; SDAI, Simplified Disease Activity Index.

Supplemental figure 9 The proportion of patients who achieved CDAI ≤2.8 in FINCH 4 according to parent study: FINCH 1 (A), FINCH 2 (B) and FINCH 3 (C) (safety analysis set, OC)

A

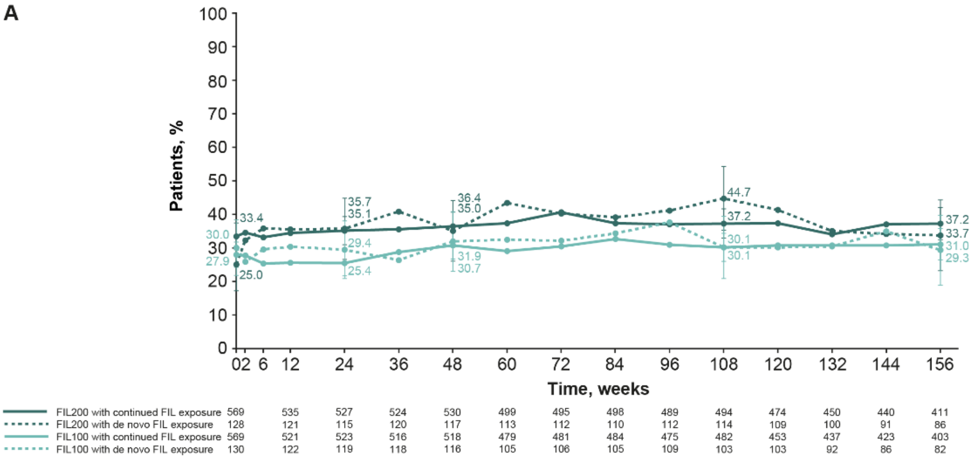

B

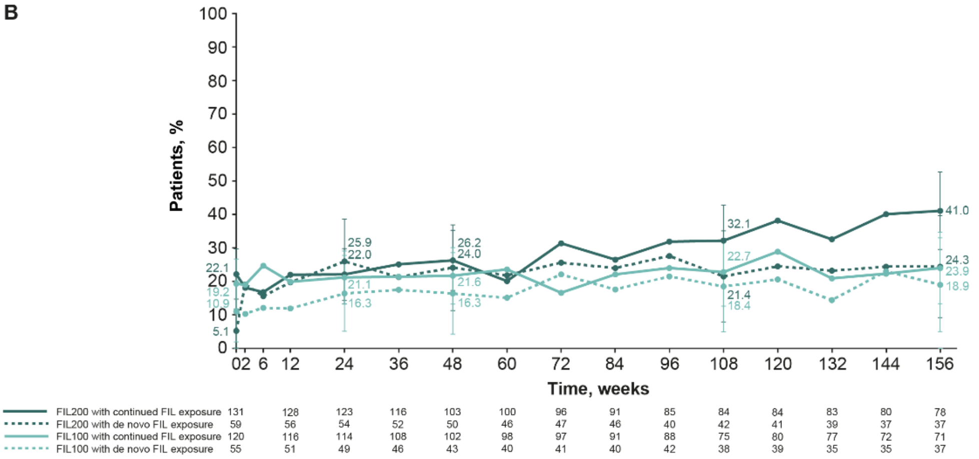

C

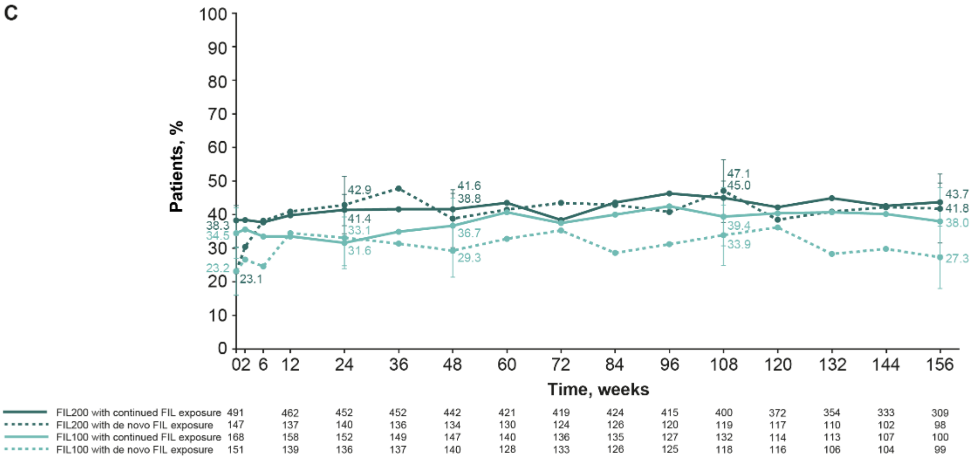

Error bars show 95% CIs.

CDAI, Clinical Disease Activity Index; FIL(100/200), filgotinib (100 mg/200 mg);

OC, observed case.

Supplemental figure 10 The proportion of patients who achieved SDAI ≤3.3 in FINCH 4 according to parent study: FINCH 1 (A), FINCH 2 (B) and FINCH 3 (C) (safety analysis set, OC)

A

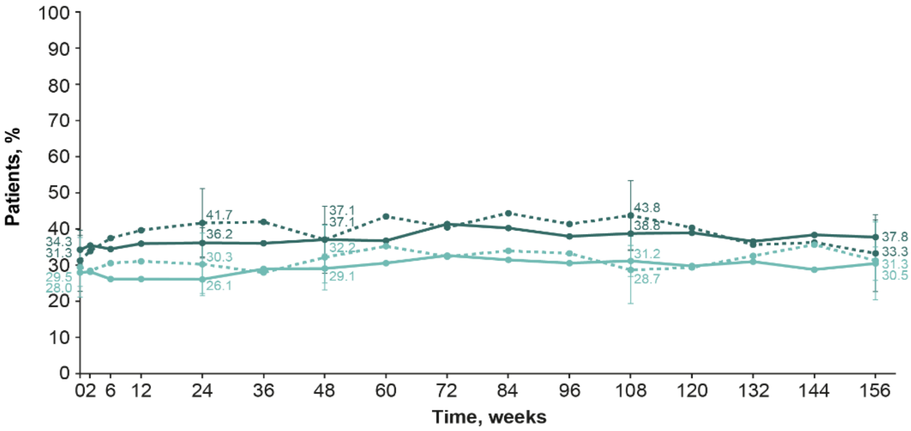

FIL200 with continued FIL exposure	569	533	525	521	529	486	481	477	479	485	461	443	430	400
FIL200 with de novo FIL exposure	128	121	115	119	116	108	111	108	111	112	109	98	91	84
FIL100 with continued FIL exposure	565	515	522	513	515	457	462	463	464	465	446	426	417	397
FIL100 with de novo FIL exposure	129	122	119	118	115	102	102	100	108	101	102	89	84	80

B

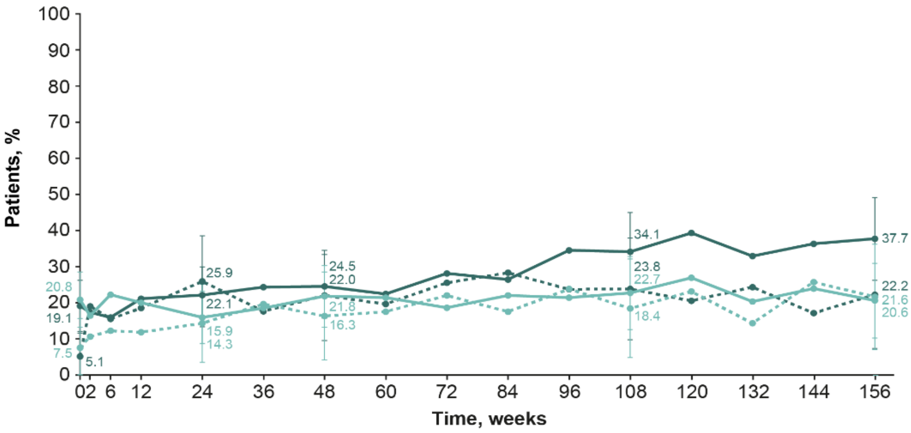

FIL200 with continued FIL exposure	131	128	122	115	102	98	96	91	84	82	84	82	80	77
FIL200 with de novo FIL exposure	59	54	54	51	50	46	47	46	38	42	39	37	35	36
FIL100 with continued FIL exposure	120	115	113	108	101	98	97	91	84	75	78	74	71	68
FIL100 with de novo FIL exposure	53	51	49	46	43	40	41	40	42	38	39	35	35	37

C

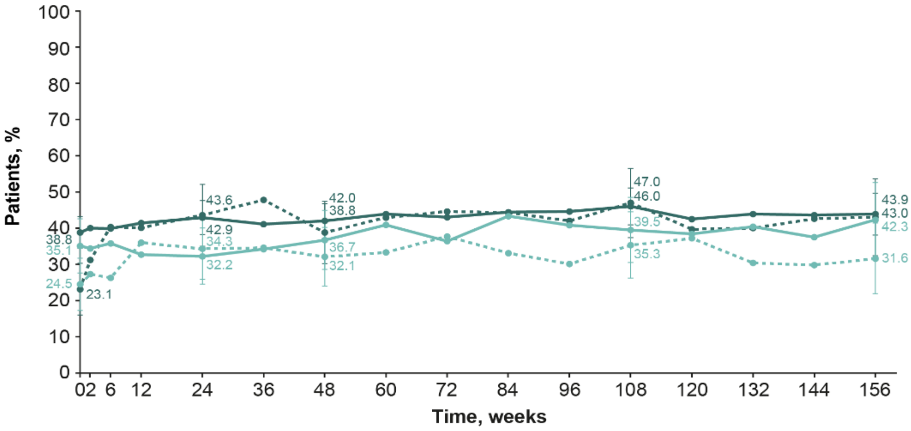

FIL200 with continued FIL exposure	490	459	450	443	441	403	393	412	406	389	367	346	328	301
FIL200 with de novo FIL exposure	147	137	140	136	134	126	121	122	119	115	116	110	101	93
FIL100 with continued FIL exposure	168	156	152	149	147	137	129	120	125	124	112	109	104	97
FIL100 with de novo FIL exposure	151	139	134	136	140	123	122	121	123	116	113	102	104	98

Error bars show 95% CIs.

FIL(100/200), filgotinib (100 mg/200 mg); OC, observed case; SDAI, Simplified Disease Activity Index.

Supplemental figure 11 Change from baseline in pain in FINCH 4 according to parent study: FINCH 1 (A), FINCH 2 (B) and FINCH 3 (C) (safety analysis set)

A

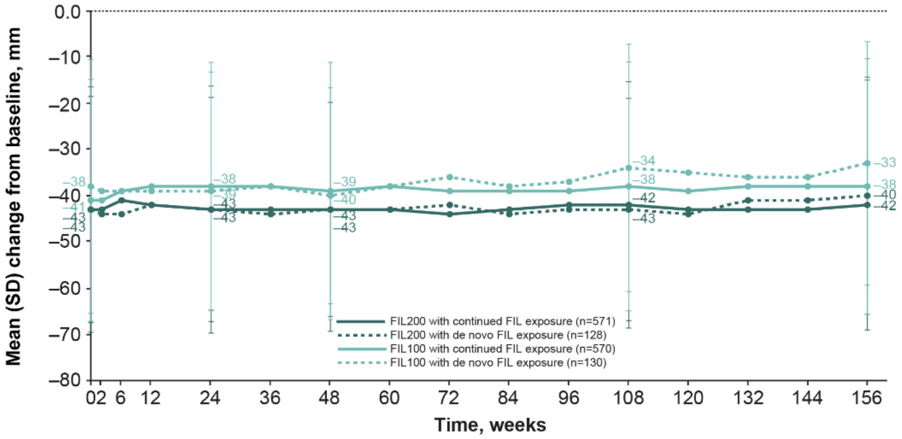

FIL200 with continued FIL exposure	569	539	529	528	536	505	499	502	493	498	475	451	440	414
FIL200 with de novo FIL exposure	128	122	118	121	118	113	112	115	114	114	109	101	92	87
FIL100 with continued FIL exposure	570	528	528	523	524	492	486	490	480	484	459	441	426	405
FIL100 with de novo FIL exposure	130	122	119	119	117	107	106	107	109	105	103	94	88	83

B

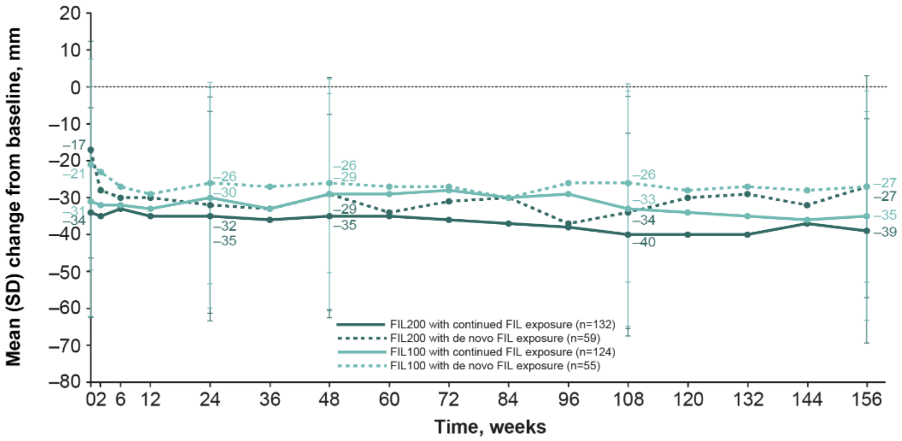

FIL200 with continued FIL exposure	132	128	123	118	105	102	98	91	87	87	84	83	80	80
FIL200 with de novo FIL exposure	59	58	56	53	50	46	47	46	41	43	42	42	38	38
FIL100 with continued FIL exposure	123	116	116	109	103	98	99	96	88	80	81	78	75	74
FIL100 with de novo FIL exposure	55	51	49	46	44	42	44	40	42	39	39	36	35	38

C

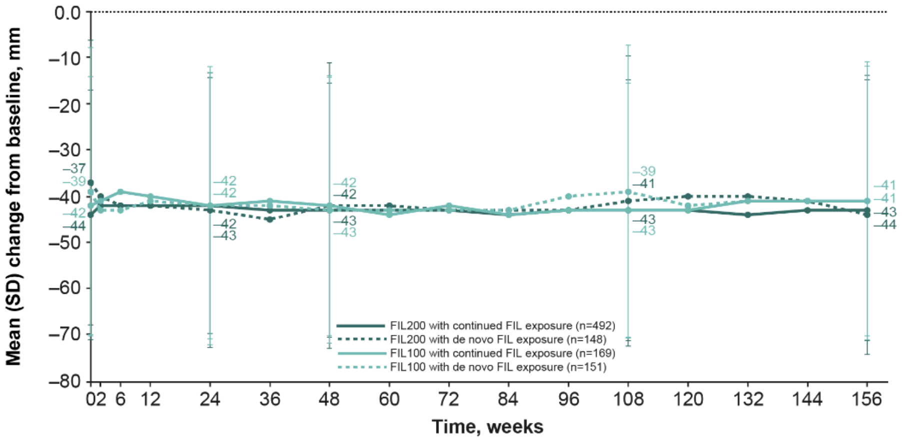

FIL200 with continued FIL exposure	491	466	461	457	445	425	425	432	419	402	381	358	335	312
FIL200 with de novo FIL exposure	147	140	141	138	136	131	124	126	121	120	118	111	103	100
FIL100 with continued FIL exposure	168	160	154	152	148	141	137	137	128	132	114	113	108	100
FIL100 with de novo FIL exposure	151	141	137	138	140	132	134	128	127	120	116	108	104	100

Baseline value was the last available value collected on or prior to the first dose of study drug. Error bars show SD.

FIL(100/200), filgotinib (100 mg/200 mg).

Supplemental figure 12 Change from baseline in HAQ-DI in FINCH 4 according to parent study: FINCH 1 (A), FINCH 2 (B) and FINCH 3 (C) (safety analysis set)

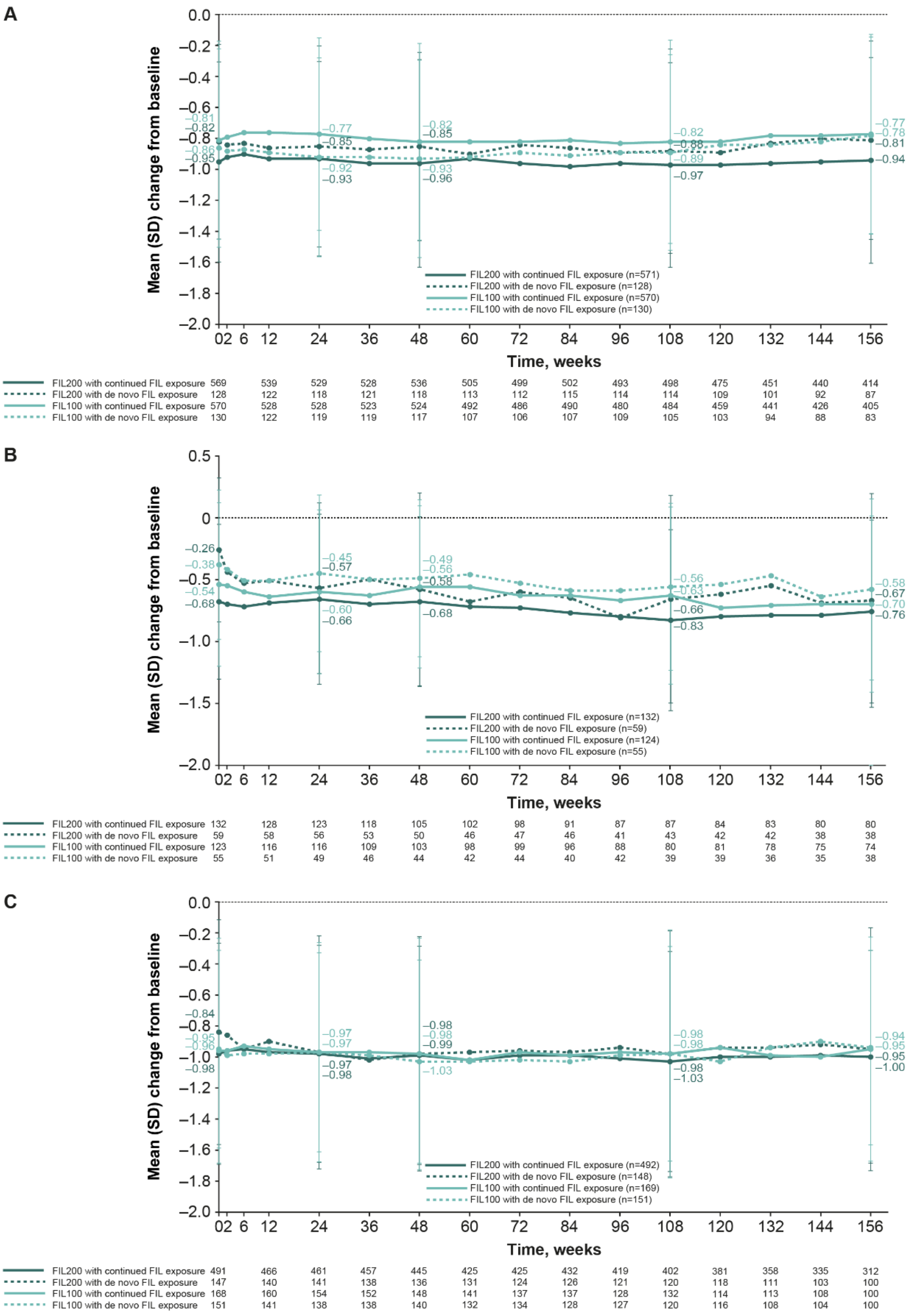

Baseline value was the last available value collected on or prior to the first dose of study drug. Error bars show SD.

FIL(100/200), filgotinib (100 mg/200 mg); HAQ-DI, Health Assessment Questionnaire–Disability Index.

Supplemental figure 13 The proportion of patients who achieved Boolean remission 1.0 in FINCH 1 (A), FINCH 2 (B) and FINCH 3 (C) (safety analysis set, OC)

A

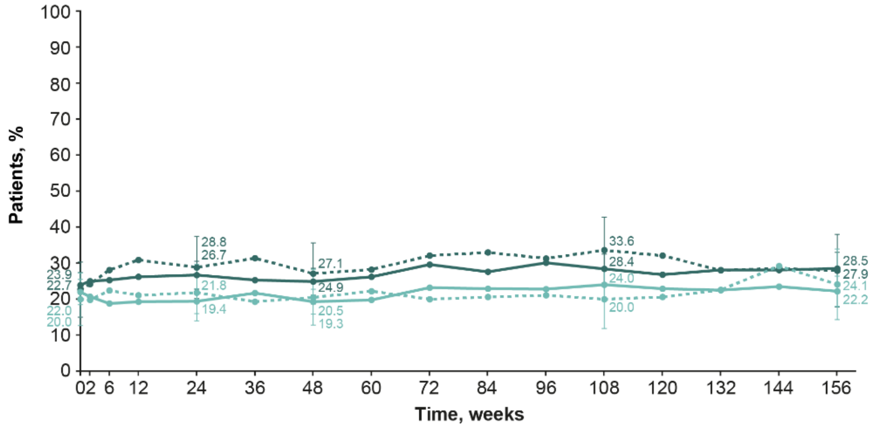

FIL200 with continued FIL exposure	570	545	536	533	535	500	494	492	489	500	474	452	441	411
FIL200 with de novo FIL exposure	128	123	118	121	118	110	112	112	112	113	109	100	91	86
FIL100 with continued FIL exposure	569	538	530	525	522	489	482	485	478	480	459	436	426	405
FIL100 with de novo FIL exposure	130	123	119	119	117	108	105	107	109	105	102	93	89	83

B

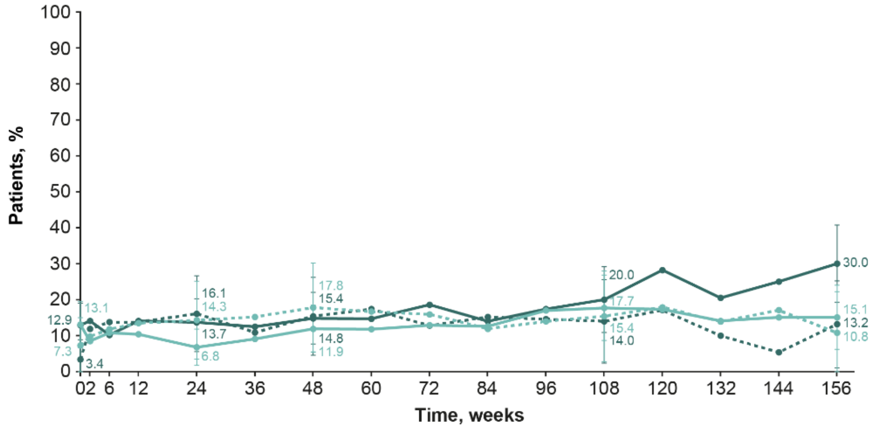

FIL200 with continued FIL exposure	132	128	124	120	108	102	97	93	86	85	85	83	80	80
FIL200 with de novo FIL exposure	59	58	56	55	52	46	47	46	41	43	41	40	37	38
FIL100 with continued FIL exposure	122	115	117	110	109	102	101	95	88	79	81	78	73	73
FIL100 with de novo FIL exposure	55	52	49	46	45	42	44	42	43	39	39	36	35	37

C

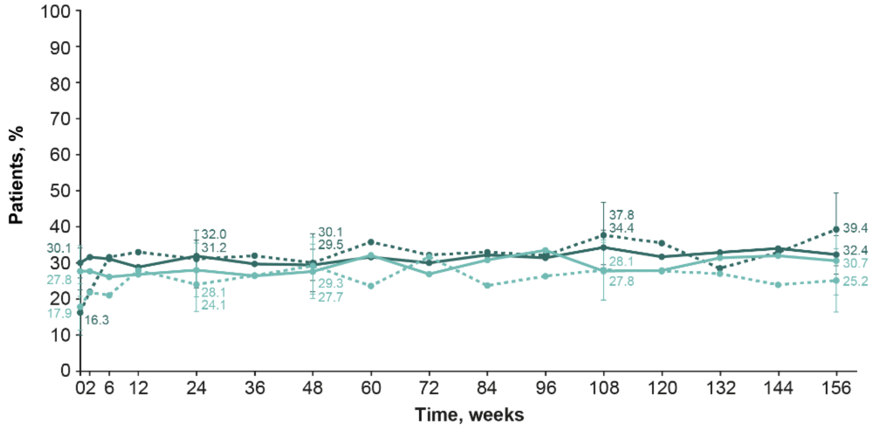

FIL200 with continued FIL exposure	492	467	465	457	447	420	418	427	416	401	381	355	334	309
FIL200 with de novo FIL exposure	147	142	141	140	136	131	124	127	121	119	118	112	103	99
FIL100 with continued FIL exposure	169	160	153	155	148	143	137	136	128	133	118	111	106	101
FIL100 with de novo FIL exposure	151	143	137	139	140	131	132	126	129	121	115	107	104	103

Error bars show 95% CIs.

FIL(100/200), filgotinib (100 mg/200 mg); OC, observed case.

Supplemental figure 14 The proportion of patients who achieved Boolean remission 2.0 in FINCH 1 (A), FINCH 2 (B) and FINCH 3 (C) (safety analysis set, OC)

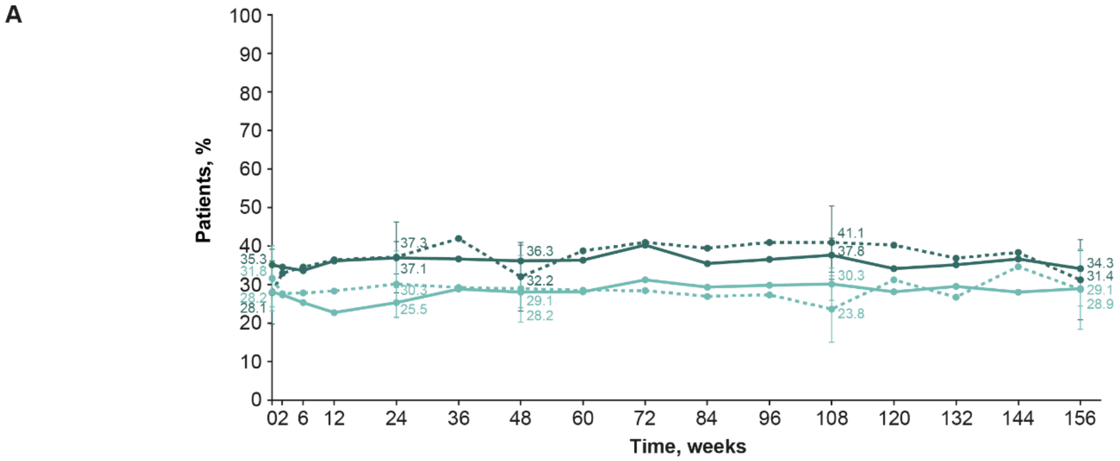

	0	12	24	36	48	60	72	84	96	108	120	132	144	156
FIL200 with continued FIL exposure	570	545	536	533	535	496	492	491	488	500	472	451	440	411
FIL200 with de novo FIL exposure	128	123	118	121	118	108	112	111	112	112	109	100	91	86
FIL100 with continued FIL exposure	568	536	529	524	522	484	481	485	476	479	459	434	426	405
FIL100 with de novo FIL exposure	129	123	119	119	117	108	105	107	109	105	102	93	89	83

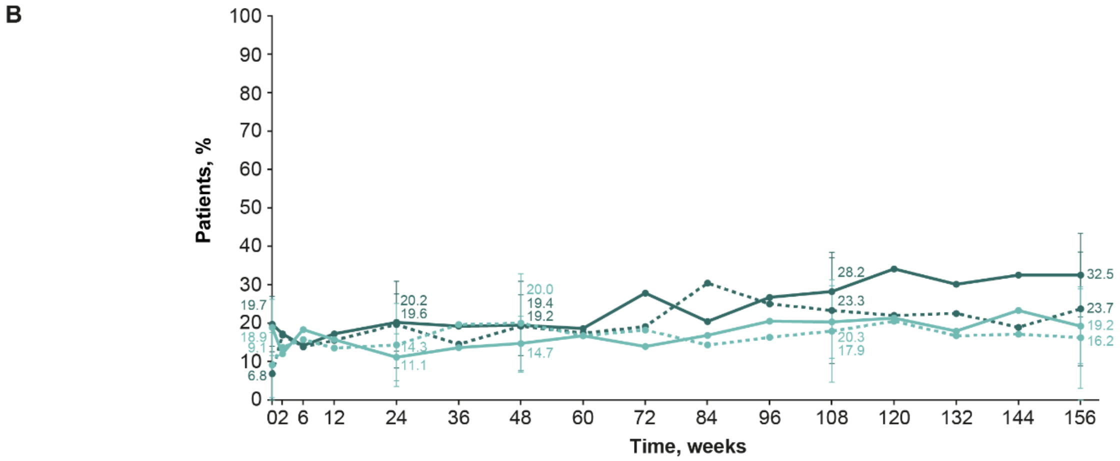

	0	12	24	36	48	60	72	84	96	108	120	132	144	156
FIL200 with continued FIL exposure	132	128	124	120	108	102	97	93	86	85	85	83	80	80
FIL200 with de novo FIL exposure	59	58	56	55	52	46	47	46	40	43	41	40	37	38
FIL100 with continued FIL exposure	122	115	117	110	109	102	101	95	88	79	80	78	73	73
FIL100 with de novo FIL exposure	55	52	49	46	45	42	44	42	43	39	39	36	35	37

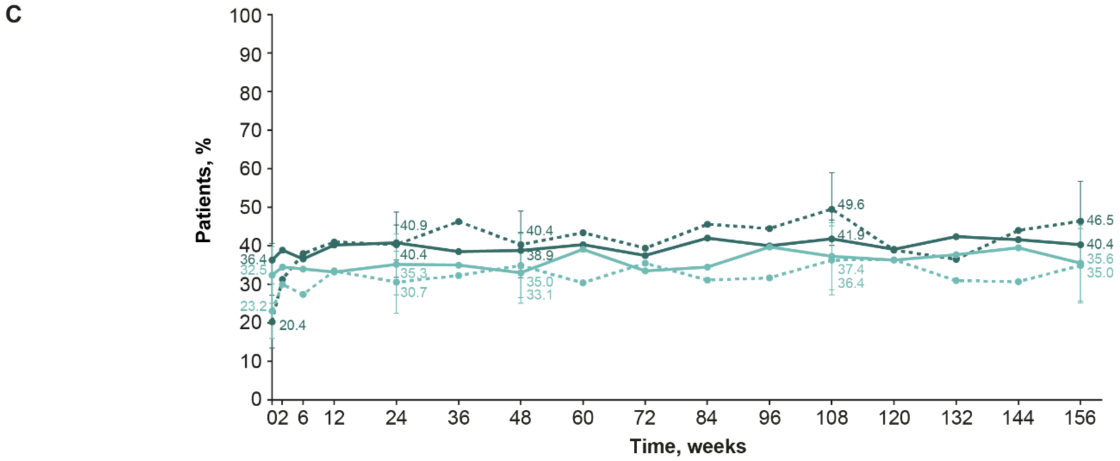

	0	12	24	36	48	60	72	84	96	108	120	132	144	156
FIL200 with continued FIL exposure	492	467	464	456	447	418	417	425	416	399	378	353	333	307
FIL200 with de novo FIL exposure	147	141	141	140	136	131	124	127	121	119	118	112	102	99
FIL100 with continued FIL exposure	169	159	153	154	148	143	137	136	128	131	118	111	106	101
FIL100 with de novo FIL exposure	151	143	137	139	140	131	132	125	129	121	115	106	104	103

Error bars show 95% CIs.

FIL(100/200), filgotinib (100 mg/200 mg); OC, observed case.
