# Supplementary material for: Efficacy and safety of filgotinib in patients with rheumatoid arthritis: week 156 interim results from a long-term extension study
Source: RMD Open. 2024 Oct 24;10(4):e004476. doi: 10.1136/rmdopen-2024-004476 (PMC11529685; doi:10.1136/rmdopen-2024-004476)
Supplement: online supplemental file 2 [file rmdopen-10-4-s002.pdf]

Efficacy and safety of filgotinib in patients with rheumatoid arthritis:

Week 156 interim results from a long-term extension study

FINCH 4 is an ongoing long-term extension study of filgotinib in patients with RA.
The primary outcome is safety and tolerability; the secondary outcome is efficacy

Patients continue to receive:

filgotinib 200 mg or **filgotinib 100 mg**

from the parent study (FINCH 1, 2 and 3) or receive filgotinib 200 mg or 100 mg de novo

Key findings

In patients with an inadequate response to methotrexate (from **FINCH 1**)

Proportion of responders following filgotinib treatment at week 156 (NRI analysis)

	ACR20 response at week 156	DAS28-CRP <2.6 at week 156	CDAI ≤2.8 at week 156	Boolean remission 1.0 at week 156	Boolean remission 2.0 criteria applied
200 mg de novo	60.2	39.1	22.7	18.8	21.1
100 mg de novo	54.6	29.2	18.5	15.4	18.5
200 mg continuous	67.3	44.3	26.8	20.5	24.7
100 mg continuous	59.5	37.9	21.9	15.8	20.7

Similar efficacy data were seen for methotrexate naïve patients (from **FINCH 3**) and patients with an inadequate response to bDMARDs (from **FINCH 2**)

No unexpected safety signals were observed

In filgotinib **200 mg** and **100 mg** groups, respectively:

EAIR per 100 PYE (95% CI)

	TEAEs: filgotinib 200 mg	TEAEs: filgotinib 100 mg	Serious TEAEs: filgotinib 200 mg	Serious TEAEs: filgotinib 100 mg
Upper CI	98.9	97.1	7.9	8.1
EAIR	93.7	91.3	7.0	7.1
Lower CI	88.7	85.8	6.3	6.2

Conclusion

filgotinib 200 and **100** mg demonstrated sustained efficacy up to week 156 in **FINCH 4**, in patients enrolled from FINCH 1, 2 or 3 with **no unexpected safety signals**

ACR20, American College of Rheumatology 20; bDMARD, biologic disease-modifying anti-rheumatic drug; CDAI, Clinical Disease Activity Index; CI, confidence interval; DAS28-CRP, Disease Activity Score 28 using C-reactive protein; EAIR, exposure-adjusted incident rate; NRI, non-responder imputation; PYE, patient-years of exposure; RA, rheumatoid arthritis; TEAE, treatment-emergent adverse event
